# Biomedical Photoacoustic Imaging for Molecular Detection and Disease Diagnosis: “Always‐On” and “Turn‐On” Probes

**DOI:** 10.1002/advs.202202384

**Published:** 2022-06-30

**Authors:** Yun Zeng, Taotao Dou, Lei Ma, Jingwen Ma

**Affiliations:** ^1^ School of Life Science and Technology Xidian University and Engineering Research Center of Molecular and Neuro Imaging Ministry of Education Xi'an Shaanxi Province 710126 P. R. China; ^2^ International Joint Research Center for Advanced Medical Imaging and Intelligent Diagnosis and Treatment and Xi'an Key Laboratory of Intelligent Sensing and Regulation of trans‐Scale Life Information School of Life Science and Technology Xidian University Xi'an Shaanxi Province 7100126 P. R. China; ^3^ Neurosurgery Department Ninth Affiliated Hospital of Medical College of Xi'an Jiaotong University Xi'an Shaanxi Province 710054 P. R. China; ^4^ Vascular Intervention Department Ninth Affiliated Hospital of Medical College of Xi'an Jiaotong University Xi'an Shaanxi Province 710054 P. R. China; ^5^ Radiology Department CT and MRI Room Ninth Affiliated Hospital of Medical College of Xi'an Jiaotong University Xi'an Shaanxi Province 710054 P. R. China

**Keywords:** biomedical imaging, diagnosis, nanoparticles, photoacoustic imaging, probes

## Abstract

Photoacoustic (PA) imaging is a nonionizing, noninvasive imaging technique that combines optical and ultrasonic imaging modalities to provide images with excellent contrast, spatial resolution, and penetration depth. Exogenous PA contrast agents are created to increase the sensitivity and specificity of PA imaging and to offer diagnostic information for illnesses. The existing PA contrast agents are categorized into two groups in this review: “always‐on” and “turn‐on,” based on their ability to be triggered by target molecules. The present state of these probes, their merits and limitations, and their future development, is explored.

## Graphical abstract

Photoacoustic imaging is a nonionizing, noninvasive imaging technique that combines optical and ultrasonic imaging modalities to provide images with excellent contrast, spatial resolution, and penetration depth. Exogenous photoacoustic contrast agents are created to increase its sensitivity and specificity to offer diagnostic information. The present state of these probes, their merits and limitations, and their future development is explored.

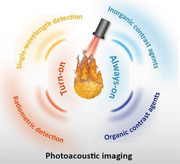

## 1 Introduction

Photoacoustic (PA) imaging is a nonionizing, noninvasive imaging technique that combines optical and ultrasonic imaging modalities. When tissue is irradiated with a pulsed laser, endogenous or exogenous contrast agents convert the energy of light to heat, resulting in an instantaneous increase in local temperature, followed by the thermoelastic expansion of tissue, which generates acoustic waves that can be collected using an ultrasound transducer and converted into PA images using data processing according to their arrival times.^[^ ^1^ ^]^ In comparison to optical and ultrasound imaging, PA imaging combines the strong contrast of optical imaging with the high spatial resolution of acoustic imaging and has attracted considerable interest in recent years.^[^ ^2^, ^3^ ^]^ PA imaging has been extensively adopted in clinical practice for imaging the breast, skin, vascular, musculoskeletal, gastrointestinal, and adipose tissue, among other situations. Endogenous contrast agents (oxyhemoglobin/deoxyhemoglobin, melanin, lipids) and genetic encoding reporters aid in the diagnosis of clinical PA imaging.^[^ ^4^, ^5^ ^]^ For example, hemoglobin provides for the monitoring of oxygen saturation; melanin absorbance allows for the monitoring of primary melanoma; particular absorbance of lipids allows for the visualization of lipid distribution in vivo.^[^ ^6^ ^]^ Although endogenous contrast agents such as hemoglobin can generate a PA imaging signal, most tissues have low near‐infrared (NIR) absorbance and hence cannot provide further tissue information in the absence of exogenous contrast agents. PA imaging using genetically encoded imaging agents can efficiently represent the distribution of labeled cells in vivo as well as cellular activity, proliferation, and expression of several intracellular markers. For genetically encoded PA imaging, melanin expression‐related tyrosinase, the bacteriophytochrome‐based NIR fluorescent protein, and the reversibly switchable bacterial phytochrome have been developed.^[^ ^7^, ^8^, ^9^, ^10^ ^]^ However, the limited imaging depth and low absorbance of endogenous PA contrast agents allow for only a limited amount of information to be obtained. As a result, it is critical to produce exogenous contrast agents that are both sensitive and specific.

The optimal PA contrast agent should have the following characteristics: 1) high extinction coefficient and photothermal conversion efficiency (PCE), 2) maximum absorbance at a specific wavelength to ensure PA detection at low concentrations, 3) absorbance in the NIR region to avoid the strong absorbance of intrinsic chromophores and thus increase deep tissue penetration, 4) high photostability, and 5) superior biosafety and metabolizability. Due to the fact that both PA imaging and photothermal therapy (PTT) are based on photothermal conversion, a variety of PA contrast agents have been developed on the basis of PTT agents, including cyanine dyes,^[^ ^11^ ^]^ organic semiconductor polymer nanoparticles (SPNs),^[^ ^12^ ^]^ gold nanorods (AuNRs),^[^ ^13^ ^]^ noble metal nanomaterials,^[^ ^14^ ^]^ carbon nanomaterials,^[^ ^15^ ^]^ and transition metal sulfides.^[^ ^16^ ^]^ Currently, the majority of established PA contrast agents may accumulate passively or actively in tumors or tissues whose PA intensities are concentration‐dependent, hence increasing PA intensity in vivo, which is referred to as “always‐on” PA contrast agents. These probes are sensitive to being disrupted by background signals and have an insufficient signal‐to‐noise (S/N) ratio. To further improve the S/N ratio, researchers developed “turn‐on” contrast agents whose absorbance spectra changed when they interacted with targeted molecules, and dynamic PA imaging could be used to monitor biomarkers or enzymes associated with pathological changes, potentially aiding in the early diagnosis of diseases.^[^ ^17^ ^]^

**Scheme 1:**
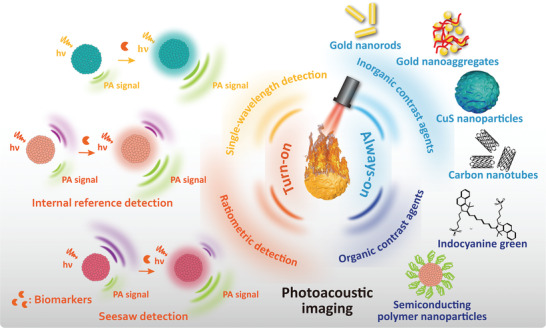
Schematic illustration of “always‐on” and “turn‐on” PA contrast agents in bioimaging.

In this review, we summarized the exogenous PA contrast agents that have been published in the last five years and categorized them into two classes: “always‐on” and “turn‐on” (**Scheme** **1**). First, we evaluated the benefits and drawbacks of “always‐on” PA contrast agents. Then, we classified “turn‐on” PA contrast agents into “single‐wavelength detection” and “ratiometric detection” PA contrast agents, and the “ratiometric detection” PA contrast agents were categorized as “internal reference detection” or “seesaw detection.”^[^ ^18^ ^]^ Finally, the current state and future prospects for PA contrast agents were presented to generate innovative ideas for the future of PA imaging in semiquantitative and quantitative methods for disease diagnosis and progression monitoring in vitro or in vivo.

## 2 “Always‐On” PA Contrast Agents

“Always‐on” PA contrast agents often have a high extinction coefficient and PCE, but their PA intensities are hardly affected by the external environment. The PA intensities of “always‐on” contrast agents have a linear relationship with their concentration. After retention at lesion areas in vivo, the concentration increase could induce PA intensity increase to assist disease diagnosis and therapy using PA imaging. For example, the high tumor accumulation of PA nanoprobes enables tumor imaging‐guided treatment. These “always‐on” PA contrast agents were summarized in **Table** **1**.

**Table 1:** Summary of “always‐on” PA contrast agents in biomedical imaging

Materials	Chromophores	*λ*_max_	Extinction coefficient	PCE	PA imaging wavelengths	Size	Applications	Refs.
LDGI	AuNRs (40 ± 5 nm × 8 ± 3 nm)	830 nm	Not mentioned	Not mentioned	820 nm	160 ± 10 nm	LDGI‐loaded MSCs for PA imaging, targeted PTT, and chemotherapy	[23]
M‐AuHNRs	Miniature hollow AuNRs	700 nm	Not mentioned	34%	1064 nm	≈46.1 nm in length and 24.7 nm in outer diameter.	PA imaging	[25]
HA‐4‐ATP‐AuNFs‐DOX	Gold nanoframework	Shifted from 750 to 1150 nm	26.2 L g^−1^ cm^−1^	23.9% (1064 nm)	1200 nm	140.2 ± 3.2 nm	PA‐Raman dual image‐guided photo‐chemotherapy	[28]
PDC/P@HCuS	Hollow mesoporous CuS NPs	1060 nm	Not mentioned	Not mentioned	Not mentioned	≈100 nm hollow structure	Fluorescence and PA images for chemo‐phototherapy	[37]
CuFeSe_2_ NCs	CuFeSe_2_ ternary nanocrystals	Not mentioned	5.8 L g^−1^ cm^−1^ (808 nm)	82%	Not mentioned	20.4 nm	Multimodal imaging	[41]
U‐BSHM	Bi_2_S_3_	Not mentioned	Not mentioned	26.8% (808 nm)	700, 800, 900 nm	280 nm for the hollow interior	PA imaging and chemo/PTT	[46]
Metallic 1T‐phase MoS_2_ nanodots	Metallic 1T‐phase MoS_2_ nanodots	Not show characteristic absorption peaks	25.6 L g^−1^ cm^−1^	43.3%	1280 nm	≈5 nm, 0.8 nm single‐layer thickness	PA imaging‐guided PTT	[48]
HMCS‐PEG‐GA	Hollow mesoporous carbon spheres	Not mentioned	Not mentioned	47.3%	715 nm	≈173 nm	PA imaging‐guided PTT	[54]
MTMPPPCAs	ICG and SQ650	SQ650 (660 nm) ICG (780 nm)	Not mentioned	Not mentioned	660/780 nm	169.3 ± 5.6 nm	Visualize breast cancer intratumor heterogeneity	[61]
T‐FBM NPs	IR780	≈780 nm	Not mentioned	Not mentioned	808 nm	≈320 nm	PA imaging and antithrombotic therapy	[73]
COF‐366 NPs	Tetra (p‐amino‐phenyl) porphyrin	Not mentioned	Not mentioned	15.07%	Not mentioned	100 nm	PA imaging‐guided PDT and PTT	[80]
TPC‐SS NPs	Chlorin dimers	650 nm	99 047 m ^−1^ cm^−1^	37%	680 nm	148 nm	PA imaging and PTT	[81]
ZnPc‐NDs	ZnPc	Characteristic Soret band (310–500 nm) and Q‐band (675 nm)	Not mentioned	45.7%	Not mentioned	80 nm	PA imaging and PTT	[87]
PcS4‐PcN4	PcS4‐PcN4	Q band	Not mentioned	Not mentioned	697 nm, 800 nm	30–100 nm	PA imaging and PTT	[89]
GdPc	GdPc	680 ​nm	​31 234 ​m ^− 1^ cm^−1^	Not mentioned	680 ​nm	130​ ± ​10 ​nm at pH 7.4 to 10 ​±​2.0​ nm at pH 5.0	MR/PA imaging‐guided parallel photocavitation and photodynamic oxidation	[91]
BODIPYsome vesicles	Aza‐BODIPY	From 695 to 702 nm	128 mm ^−1^ cm^−1^	Not mentioned	702 nm	80–102 nm	PA/fluorescence imaging	[98]
TB1 dots	TB1	352 and 710 nm	10.2 L g^−1^ cm^−1^ (740 nm)	Not mentioned	740 nm	≈36 nm	NIR‐II fluorescence and NIR‐I PA imaging of orthotopic brain tumors	[99]
cRGD‐PDI NPs	Perylene‐3,4,9,10‐tetracarboxylic diimide	A maximum absorption at 650 nm and a shoulder at 700 nm	2.58 × 10^8^ m ^−1^ cm^−1^ (700 nm)	Not mentioned	700 nm	41.2 ± 2.5 nm	Lightening early thrombus and monitoring thrombolysis in living mice	[102]
QDI‐NPs	QDI	≈700 nm	1.3 × 10^5^ m ^−1^ cm^−1^	64.7 ± 4%	800 nm	10.8 ± 1.4 nm	PA imaging and PTT	[103]
P(DPP‐BT/DOX) NPs	DPP‐BT	686 nm	Not mentioned	50.0%	730 nm	≈60 nm	NIR‐II fluorescence/PA imaging‐guided PTT/PDT/chemotherapy	[105]
SPA2PEG2	SPNs	616 and 665 nm	Not mentioned	Not mentioned	680 nm	≈25 nm	PA imaging	[112]
P1RGD NPs	P1 molecules	1064 nm	22.6 L g^−1^ cm^−1^	30.1%	1064 nm	≈50 nm	PA imaging guide NIR‐II PTT	[114]
PFTDPP‐SNAP NPs	PFTDPP	550–900 nm	Not mentioned	48%	808 nm	52 nm	NIR‐II/PA imaging‐guided photothermal initiated nitric oxide/PTT	[115]
CP3 NPs	CP3 NPs	783 nm	57.7 L g^−1^ cm^−1^ (under 808 nm)	Not mentioned	780 nm	43 to 52 nm	PA/PTT	[124]
SiO_2_‐CS@PPy‐PDA NPs	PPy‐PDA hybrid	≈650 nm	Not mentioned	40.7%	700 nm	PPy‐PDA hybrid (≈10 nm) onto a SiO_2_ nanoparticle (≈100 nm)	Raman and PA imaging	[133]
PANI‐ES@AOT	Polyaniline	≈1000 and ≈420 nm	26.3 L g^−1^ cm^−1^ (1064 nm)	43.9%	970 nm	From 77.8 ± 0.7 nm (pure vesicles); 78.5 ± 0.5 nm (no H_2_O_2_ control); 82.3 ± 0.7 nm (after the reaction)	PA imaging guided PTT‐chemotherapy in NIR‐II region	[136]

### 2.1 Inorganic PA Contrast Agents

#### 2.1.1 Gold Nanomaterials

Due to the localized surface plasmon resonance (LSPR) phenomenon, gold nanomaterials exhibit NIR photothermal properties. When exposed to a laser, their conduction electrons oscillate relative to the core, further converting light to the PA signal. Their high absorbance cross‐section, stability, bioinertness, and maximal absorbance wavelength can be easily modified by tuning their surface‐to‐volume ratio, polarization mode, edge/vertex count, and “sharpness,” making them effective PA contrast agents in vivo.^[^ ^19^ ^]^ Until now, a range of gold nanomaterials (nanorods, nanocages, nanostars, nanoshells, and nanobipyramids) and gold nanoassemblies have been offered for PTT and PA imaging in the NIR region. One of the primary mechanisms for surface modification is to form a strong gold–sulfur bond to modify gold nanomaterials.

##### AuNRs

**Figure 1:**
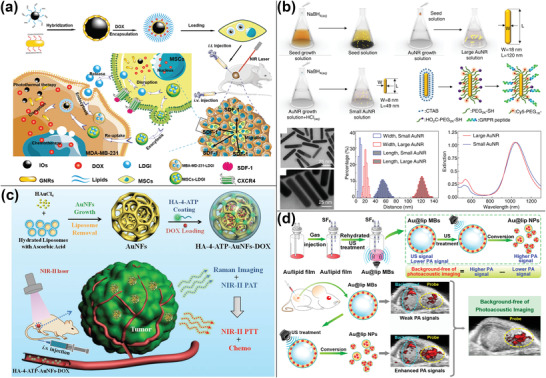
PA imaging using gold nanomaterials. a) Detection of MSCs labeled with liposomes containing AuNRs, iron oxide, and DOX using PA imaging. Reproduced under the terms of the Creative Commons CC‐BY license.^[^ ^23^ ^]^ Copyright 2018, The Authors. Published by Wiley‐VCH. b) Miniature AuNRs for PA imaging in the NIR‐II range. Reproduced with permission.^[^ ^24^ ^]^ Copyright 2019, Springer Nature. c) Raman reporter gold nanoshells loading 4‐aminothiophenol and DOX for PA‐Raman imaging‐guided chemo/PTT. Reproduced with permission.^[^ ^28^ ^]^ Copyright 2020, Wiley‐VCH. d) Ultrasound‐responsive microbubbles combined with gold nanoclusters enabling PA imaging with no background signal. Reproduced with permission.^[^ ^35^ ^]^ Copyright 2019, American Chemical Society.

AuNRs are one of the most frequently employed photothermal and PA imaging agents; their absorbance in the NIR range is mostly due to longitudinal surface plasmon excitations, with their maximal absorbance proportional to their aspect ratio. The absorbance peak is substantially redshifted as the aspect ratio increases, and so may be adjusted by altering their aspect ratio.^[^ ^20^ ^]^ AuNRs’ high PCE (22.6–32%) makes them ideal contrast agents for PA imaging in vivo, as well as nanocarriers for various imaging probes and antitumor drugs.^[^ ^13^, ^21^, ^22^ ^]^ Targeted therapy is essential for the treatment of triple‐negative breast cancer. Xu et al. coencapsulated AuNRs, iron oxide nanocluster, and doxorubicin (DOX) in liposomes prior loading them into mesenchymal stem cells (MSCs) for multimodal imaging‐guided synergetic chemo/PTT of triple‐negative breast cancer. The iron oxide nanocluster could release iron ions to increase the expression of CXC4, which was associated with MSC migration ability, whereas AuNRs could be used as a photothermal/PA imaging agent for photothermal triggering of drug release and tracing of MSCs in vivo (**Figure** **1**a).^[^ ^23^ ^]^ To shift the LSPR absorbance peak of AuNRs to correspond to NIR‐II imaging of deeper tissue penetration, their aspect ratio is tuned to around 6, resulting in their long sides exceeding 100 nm, which is unfavorable for tumor cell uptake. To resolve the contradiction, Chen et al. accurately controlled the NaBH_4_ concentration and pH values throughout the growth of AuNRs seeds, resulting in miniature AuNRs ((49 ± 8 nm) × (8 ± 2 nm)) with the same aspect ratio but a smaller size than standard AuNRs ((120 ± 17 nm) × (18 ± 4 nm)). Small AuNRs had a threefold increase in photostability, and PA signals were 3.5 and 4.5 times stronger than normal ones both in vitro and in vivo (Figure 1b).^[^ ^24^ ^]^ Cai et al. referenced the plasmonic sub‐hybridization process of AuNRs and nanocages and used TeSe nanorods as sacrificial templates to manufacture hollow AuNRs. In tumors, hollow AuNRs had a high absorbance and PA intensity at 1064 nm.^[^ ^25^ ^]^ However, AuNRs have certain drawbacks, including narrow absorbance peaks that are often concentrated in the NIR‐I region with limited tissue penetration depth and a fabrication procedure that involves a toxic surfactant such as cetyltrimethyl ammonium bromide (CTAB). To increase their biocompatibility, shells such as polydopamine (PDA),^[^ ^26^ ^]^ and mesoporous silica,^[^ ^27^ ^]^ were selected to cover AuNRs. These shells not only lessen AuNRs’ toxicity but also provide additional imaging probes and chemotherapy medications.

##### Gold Nanoshells and Nanocages

The absorbance maximum of gold nanoshells and nanocages may be modified by varying the thickness or size of the shell layer. Gold nanoshells with a mesopore structure may be readily synthesized by in situ reductions, adsorption, and re‐crosslinking of smaller gold nanoparticles (NPs), or by employing sacrificial templates. Wang et al. created a gold nanoframework with large mesopores using liposomes as templates. The large mesoporous structure of the gold nanoframework exhibited promising NIR‐II PA imaging properties, and their formation of electromagnetic “hotspots” could amplify the surface‐enhanced Raman scattering (SERS) signals of the Raman reporter 4‐aminothiophenol, as demonstrated by finite difference time domain simulations. Additionally, the mesoporous might be used to load DOX and combat cancer (Figure 1c).^[^ ^28^ ^]^

##### Gold Nanoassemblies

LSPR produces the NIR absorbance of gold nanomaterials. To improve LSPR or to shift LSPR peaks toward the NIR region, it is required to create gold nanomaterials with certain geometries, which requires a complicated preparation procedure and the use of the hazardous surfactant CTAB. Additionally, the lack of photostability was a disadvantage, limiting the functionality accessible to applications. When gold NPs are close to each other to form assemblies, their plasma oscillations couple and the electric field enhancement between gaps of NPs are significantly greater than that between individual NPs, resulting in their LSPR absorbance redshift, an increase in the absorbance coefficient *μ*_a_ in the NIR region, and thus further enhancement of the PA imaging effect.^[^ ^2^, ^29^, ^30^ ^]^ Typically, gold nanoassemblies have been formed in vitro and in vivo; for instance, inorganic NPs such as MnO NPs,^[^ ^31^ ^]^ amphiphilic nanovesicles or micelles,^[^ ^30^, ^32^ ^]^ may self‐assemble into gold nanoassemblies to assemble gold nanoclusters, gold nanochains,^[^ ^33^ ^]^ and so on. Cheheltani et al. improved prior work by incorporating kidney‐excretory gold NPs (<5.5 nm) into biodegradable poly di(carboxylatophenoxy)phosphazene for the manufacture of gold nanoassemblies. PA signals were obtained in vivo following injection into the muscle of a mouse leg, and no additional injury was found after three months. Although the intracellular metabolism of gold nanoassemblies has been documented, the intracorporal environment has not been thoroughly investigated.^[^ ^34^ ^]^ Meng et al. took advantage of the nanovesicles’ sensitivity to ultrasound to create gold nanomaterials embedded in the lipid bilayer of liposomes conveying SF_6_ gas. After ultrasonic stimulation, the nanovesicles burst to create gold nanoclusters, increasing the S/N ratio of PA signals (Figure 1d).^[^ ^35^ ^]^

Small gold NPs modified with responsive chemical groups on their surface can be aggregated when they are triggered by tumor microenvironment or external stimulus, and then they are detained in tumors, and enhance the S/N ratio of PA imaging effectively. Cheng et al. coated 20 nm gold NPs with light‐responsive diazirine, which formed carbene when exposed to a 405 nm laser, and then covalently crosslinked them with ‐XH (X = C, N, O, S) groups. Using this technique, gold NPs were aggregated to form gold nanoassembles, enabling PA imaging in 4T1 tumor‐bearing mice. However, the wavelength of 405 nm is insufficient to penetrate tissue and is thus limited to the epidermis, limiting the functionality accessible to deep tissues.^[^ ^36^ ^]^

#### 2.1.2 Transition Metal Sulfides

##### CuS

**Figure 2:**
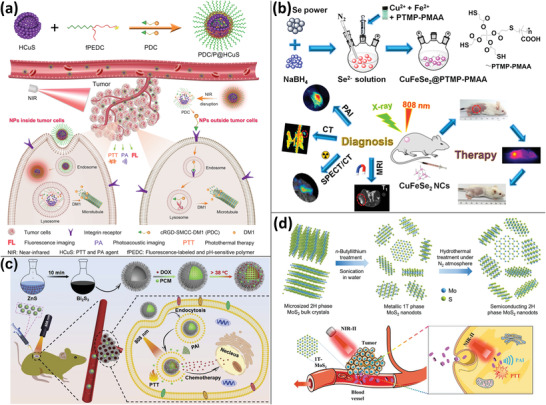
Using transition metal sulfides for PA imaging. a) Hollow mesoporous CuS NPs loading peptide‐drug conjugates for PA‐guided chemo/PTT. Reproduced with permission.^[^ ^37^ ^]^ Copyright 2019, American Chemical Society. b) Ultrasmall CuFeSe_2_ NPs for multimodal imaging‐guided PTT using PA, MRI, CT, and SPECT/CT. Reproduced with permission.^[^ ^41^ ^]^ Copyright 2017, American Chemical Society. c) Hollow Bi_2_S_3_ urchin‐like microspheres loading DOX and PCM for PA imaging‐guided PTT and temperature‐activated drug release in chemotherapy. Reproduced with permission.^[^ ^46^ ^]^ Copyright 2020, Elsevier Ltd. d) Metal 1T‐phase MoS_2_ nanodots with a narrower bandgap and improved PA imaging capability in comparison to semiconductor 2H‐phase MoS_2_. Reproduced with permission.^[^ ^48^ ^]^ Copyright 2020, Wiley‐VCH.

Due to their absorbance in the NIR region and high PCE, transition metal sulfides are of interest for PTT and PA imaging. CuS has been extensively produced in a variety of sizes and morphologies (NPs, nanosheets, nanoprisms, and hollow mesoporous nanoshells) for PA imaging‐guided tumor therapy. For instance, Sun et al. synthesized peptide‐drug conjugates (tumor‐targeted polypeptides were coupled to maytansinoid through thioether) and loaded them onto the surface of hollow mesoporous CuS NPs, resulting in triggered drug release and PA imaging in tumors (**Figure** **2**a).^[^ ^37^ ^]^ To improve the PCE of CuS NPs, ternary CuS compounds such as Gd:CuS nanotheranostic agents,^[^ ^38^ ^]^ poly(vinylpyrrolidone)–Cu–Sb–S nanotheranostic agent,^[^ ^39^ ^]^ and poly(ionic liquid)‐gated CuCo_2_S_4_^[^ ^40^ ^]^ have been created. By including more components, not only did CuS achieve a greater PCE, but it also enabled the use of other imaging modalities (e.g., magnetic resonance imaging, MRI). According to a typical example, Jiang et al. synthesized magnetic ternary nanocrystals CuFeSe_2_ with 82% PCE that could be used as a tetra‐modality imaging contrast agent (PA imaging, MRI, and computerized tomography (CT)) and as a single photon emission computed tomography (SPECT)/CT imaging contrast agent after being tagged with the radioisotope ^99m^Tc (Figure 2b).^[^ ^41^ ^]^ Additionally, coupling CuS with fluorocarbon (ultrasound contrast agents with low boiling point) is a viable way of increasing the PA signal. Santiesteban et al. coupled CuS NPs with laser‐activated perfluorinated carbon nanodroplets and developed a novel image processing technique to identify their PA signal from tissue after lowering the background signal.^[^ ^42^ ^]^

##### Ag_2_S

Ag_2_S with a narrow bandgap has been developed as a semiconductor for NIR‐II fluorescence/PA imaging. Therefore, some Ag_2_S‐based theranostic nanoplatforms have been created. For example, Ag_2_S modified with targeting ligands enhanced its accumulation in tumors and provided synergistic treatment when combined with other chemotherapeutic drugs (such as paclitaxel^[^ ^43^ ^]^ or heat shock protein inhibitors^[^ ^44^ ^]^). However, its low PCE (21–35.2%) precludes its employment as a PA contrast agent. Although it is possible to increase the PCE (58.2%) by adding additional transition metals such as Cu,^[^ ^45^ ^]^ other ways should be explored.

##### Bi_2_S_3_ and MoS_2_

Bi and Mo sulfides were also produced for PTT and PA imaging. Due to the narrow bandgap (≈1.33 eV) and high NIR absorbance of Bi_2_S_3_, Zhang et al. developed rod‐based urchin‐like hollow microspheres of Bi_2_S_3_ by core loading thermally sensitive 1‐tetradecanol (phase change material, PCM) and DOX. When the temperature was elevated beyond 38 °C, PCM induced the release of DOX, and Bi_2_S_3_ was involved in PA imaging‐guided PTT (Figure 2c).^[^ ^46^ ^]^ According to a comparable study conducted by Chen et al., MoS_2_ nanosheets with distinct layers were created utilizing an albumin‐assisted peeling approach. They discovered that single‐layer MoS_2_ nanosheets outperformed few‐layer and multilayer nanosheets in terms of PA imaging performance due to their increased light absorbance and elasticity, as well as their increased uptake of U87 glioma cells, indicating that they could be used as sensitive PA imaging‐guided PTT agents.^[^ ^47^ ^]^ Zhou et al. evaluated the PA performance of MoS_2_ in two distinct phase modulations (1T‐ and 2H‐phase). In comparison to semiconducting 2H‐phase MoS_2_ (bandgap ≈ 1.83 eV), metallic 1T‐phase MoS_2_ had a narrower bandgap, allowing PA imaging in the NIR‐II region with a PCE of 43.3%, which was much greater than 2H‐phase MoS_2_ (21.3%). After surface modification with polyvinyl pyrrolidone, 1T‐phase MoS_2_ nanodots successfully directed tumor PTT using PA imaging under 1064 nm laser irradiation (Figure 2d).^[^ ^48^ ^]^ Although transition metal sulfide semiconductors with narrow bandgaps have been produced for PA and PTT, they often have drawbacks such as poor PCE, and their degradation and metabolism in vivo are still debated.

#### 2.1.3 Carbon Nanomaterials

Carbon nanomaterials have attracted the attention of scientists as one of the most commonly employed PA imaging and photothermal agents. Carbon nanomaterials outperformed gold nanomaterials in terms of photostability and biocompatibility, as well as ease of fabrication. Carbon nanotubes and graphene oxide with sp‐ and sp^2^‐hybridized carbon atoms, in particular, have an extended *π*‐conjugation system in their structures, making them outstanding PA contrast and photothermal agents in the NIR region with high absorbance and PCE.^[^ ^49^ ^]^

Mesoporous carbon nanospheres and nanoshells created by hydrothermal or high‐temperature carbonization, as well as mesoporous carbon nanoshells prepared via template removal and carbonization, have mesoporous architectures that may be used to load chemotherapeutic medicines.^[^ ^50^, ^51^, ^52^, ^53^, ^54^ ^]^ Apart from chemotherapeutic drugs, coloading carbon nanomaterials with gas (e.g., by the formation of gas‐generating precursors or codelivery in microbubbles) is also a useful tool for increasing the PA signal of carbon nanomaterials. For example, in the study of Yu et al., CO_2_ was introduced to the neutral pillar[6]arene (CP6) tertiary amine, and subsequently, CP6 was coupled with the amphiphilic molecule PyN containing pyrene tails through host–guest contact. Then, via *π*–*π* stacking, these host–guest compounds were coloaded onto graphene oxide. The PA signal of graphene oxide in this system might be amplified by CO_2_ nanobubbles created by bicarbonate counterions when exposed to an NIR laser.^[^ ^55^ ^]^

The biocompatibility and photostability of carbon‐based nanomaterials are exceptional. Their poor biodegradability, on the other hand, restricted their applicability. To aid in their in vivo metabolism, small‐sized carbon nanodots were created. Lee et al. discovered that N‐doped carbon nanodots decomposed during lymphatic circulation as observed by sentinel lymph node imaging and were eventually eliminated through urine.^[^ ^56^ ^]^ However, carbon‐based nanomaterials face concerns about long‐term toxicity following injection due to their low biometabolizability and biodegradability; second, the preparation process requires high temperatures; and third, while their absorbance is in the NIR region, they lack absorbance peaks at specific wavelengths. Their PCE and PA imaging capabilities can be increased by coloading with other photothermal or nanobubble‐generating materials; however, their maximum absorbance wavelength cannot be modified by structural modification. Additionally, there are limited reports of PA imaging with deep tissue penetration in the NIR‐II window.

### 2.2 Organic PA Contrast Agents

#### 2.2.1 Small Organic Molecules

##### Cyanine‐Based Dyes

**Figure 3:**
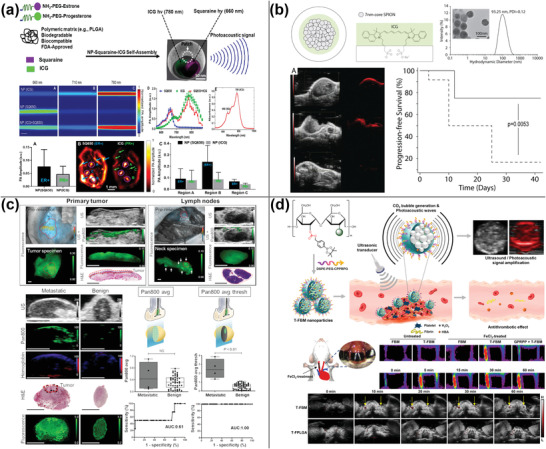
PA imaging using cyanine dyes. a) Estrogen/progesterone‐modified PEG wrapping SQ650 and ICG to detect breast cancer heterogeneity using PA imaging. Reproduced with permission.^[^ ^61^ ^]^ Copyright 2021, American Chemical Society. b) Intraoperative PA imaging using ICG‐SPIO nanoclusters to reveal tumor margins. Reproduced with permission.^[^ ^63^ ^]^ Copyright 2017, Wiley‐VCH. c) EGFR antibody modified IRDye800CW was utilized to detect lymph node metastases in head and neck squamous cell carcinoma using fluorescence and PA imaging. Reproduced with permission.^[^ ^72^ ^]^ Copyright 2021, Society of Nuclear Medicine and Molecular Imaging. d) Synthesis of thrombus‐specific recognition nanomaterials using borobenzyl carbonate and IR780 in combination with maltodextrin for PA imaging‐guided therapy of H_2_O_2_‐associated cardiovascular disease. Reproduced with permission.^[^ ^73^ ^]^ Copyright 2017, American Chemical Society.

Indocyanine green (ICG), a tricarbocyanine dye, is an FDA‐approved low‐toxicity dye for a range of clinical applications including ophthalmic imaging, hepatic function assessment, and blood flow measurement. Its fluorescence excitation and emission peaks are located at 780 and 830 nm, respectively, and it has a low fluorescence quantum yield in water (≈10%) and a high excitation energy release in nonradiative decay (90%).^[^ ^57^, ^58^ ^]^ This makes it an excellent NIR fluorescence and PA imaging contrast agent. However, certain drawbacks limit its practical applicability, including quick degradation in water, poor photostability, a short internal half‐life, and rapid clearance upon intravenous administration due to its easy binding to serum in the blood.^[^ ^59^ ^]^ Nanotechnology has emerged as a keyway of addressing these shortcomings. Humbert et al. used liposomes as carriers and molecular fluorescence/PA tomography imaging to study the mouse tibial cavity. Liposomes inhibited ICG aggregation in the bone marrow cavity, and PA tomography imaging was more sensitive, penetrated deeper tissue, and had a greater spatial resolution.^[^ ^60^ ^]^ Li et al. employed PA tomography imaging to assess the heterogeneity of breast cancer. They developed estrone and progesterone‐modified polyethylene glycol (PEG) wrapping probes with two PA signals (square amine SQ650 (660 nm) and ICG (780 nm)). In T‐47D‐implant (human breast cancer cells) tumor tissues expressing high levels of estrogen and progesterone receptors, the probes were able to effectively distinguish tissues expressing estrogen (with SQ650 as the primary signal), progesterone (with ICG as the primary signal), or both estrogen and progesterone receptors concurrently, which was consistent with immunohistochemical results. However, targeted NPs containing ICG and SQ650 leakage have been identified in vivo (**Figure** **3**a).^[^ ^61^ ^]^

While PA imaging overcomes the limitations of fluorescence imaging in terms of tissue penetration, PA imaging cannot provide whole‐body imaging. Thus, combining it with other imaging methods (e.g., fluorescence imaging, MRI) and developing multimodal imaging contrast agents may somewhat compensate for its disadvantages. Liu et al. described the use of upconversion NPs with a multishell structure coated with ICG for multimodal imaging using PA/fluorescence/MRI.^[^ ^62^ ^]^ Recurrence of a brain tumor after surgery happens as a result of the tumor's insufficient excision. Thawani et al. employed amphiphilic ICG as surfactants to transfer hydrophobic superparamagnetic iron oxide NPs into the aqueous phase, resulting in the formation of nanoclusters that might be used to guide surgical resection using preoperative MRI and intraoperative PA imaging. Finally, mice treated with imaging‐guided surgical resection had a higher survival rate than mice treated with microsurgical resection in groups (Figure 3b).^[^ ^63^ ^]^

The strength of the PA signal from an “always‐on” contrast agent is highly correlated with the concentrations of probes. As a result, increased probe accumulation in tissues is more advantageous for imaging. One successful strategy for increasing the concentration of ICG‐loaded nanomaterials in diseased locations is to modify targeting ligands on their surface. Capozza et al. conjugated targeting RGD peptide to ICG, increasing the probes’ accumulation in U‐87MG cells with high *α*_v_ *β*_3_ expression compared to A431 cells with low *α*_v_ *β*_3_ expression.^[^ ^64^ ^]^ Biological targeting is another essential method of targeting, in which A549 cell membranes disguised with ICG‐loaded PLGA encapsulating perfluorocarbon might actively target tumor tissues for homologous targeting.^[^ ^65^ ^]^ Additionally, to the approaches outlined above, combining ICG with other PA contrast agents increased their PA signal,^[^ ^66^, ^67^, ^68^, ^69^ ^]^ while encapsulating ICG in a stiff mesoporous material increased its PA signal by lowering heat conductivity and minimizing photolysis/pyrolysis.^[^ ^70^ ^]^

NIR dyes of the IR‐ and Cy‐family are likewise cyanine‐based dyes. Conjugation with targeting ligands, such as RGD, may promote their accumulation in malignancies. For example, IR820‐E[c(RGDfK)]_2_ was synthesized by combining one molecule of IR820 with two molecules of the targeting ligand RGD. This compound attached selectively to hepatocellular carcinoma (HCC) cells expressing a high level of integrin *α*_v_ *β*_3_. It demonstrated a greater PA signal in the orthotopic HCC model compared to free IR820 and effectively localized irregular HCC tissue preoperatively, delineated the tumor boundary intraoperatively, and assessed the invisible postoperative margin.^[^ ^71^ ^]^ Metastasis to lymph nodes is a significant predictive factor in individuals with head and neck squamous cell carcinoma. CT and MRI cannot detect lymph node metastases smaller than 10 mm in diameter. Nishio et al. developed targeted molecular probes by combining anti‐epidermal growth factor receptor (EGFR) antibodies with IRDye800CW. These probes were then employed in PA imaging to differentiate 53 lymph nodes with a maximum diameter of 10 mm in patients before surgery. These findings demonstrated that benign and malignant lymph nodes might be differentiated efficiently (Figure 3c).^[^ ^72^ ^]^ Coloading with gas‐generating nanomaterials may increase the PA signal through thermoelastic expansion and gas production. To overcome the poor bubble stability and short signal lifetime associated with perfluorocarbon loading carriers, Jung et al. used the interaction of glutathione carbonate with H_2_O_2_ to generate CO_2_ in H_2_O_2_‐rich thrombus, thereby avoiding the use of perfluorocarbons. The resulting CO_2_ bubbles enhanced the PA signal of IR780 (Figure 3d).^[^ ^73^ ^]^ Maltotriose are the main source of bacterial glucose and could be taken up by bacteria through the maltodextrin pathway, which does not exist in mammalian cells. By linking maltotriose to imaging probes, it is possible to discern between inflammation and bacterial infection. As a result, Zlitni et al. developed a Cy7‐1 maltotriose probe that was taken up by a variety of G+ and G− bacterial strains and PA imaging was utilized for infection detection, infection burden evaluation, and antibiotic treatment effectiveness assessment.^[^ ^74^ ^]^ Additionally, structural modifications were made to address the shortcomings of current probes. Li et al. developed a cyanine‐based probe with an asymmetric shape that enhanced its photostability and was capable of clearly indicating tiny vessels in PA imaging after BSA encapsulation.^[^ ^75^ ^]^

Cyanine‐based probes have developed into commercial dyes, most notably ICG, which is an FDA‐approved dye for clinical use. However, its poor solubility in water necessitates structural modification or nanocarrier wrapping. Additionally, their lack of photostability restricts their practical applicability. Although the encapsulation of nanocarriers mitigates some of their drawbacks, their clinical use requires further work.

##### Porphyrin‐Based Probes

Porphyrins, phthalocyanines, and naphthalocyanines are all macrocyclic compounds with four pyrrole subunits that exhibit a significant absorbance in the NIR range. Due to their poor water solubility, researchers have developed new water‐soluble derivatives based on molecular engineering or constructed theranostic nanocarriers. After loading porphyrin‐based probes into lipid bilayers, amphiphilic polymers, or mesoporous nanostructures, they may undergo aggregation‐induced quenching, resulting in nonradiative energy transfer (NRET) and PA intensity amplification. As a result, they are effective agents for combining photodynamic therapy (PDT) with PTT.

**Figure 4:**
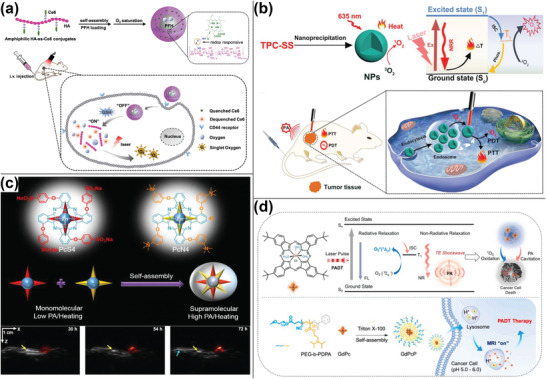
PA imaging using porphyrins and phthalocyanines analogs. a) HA‐grafted Ce6 loaded with perfluorohexane contrast agent for fluorescent, PA imaging‐guided PDT. Reproduced with permission.^[^ ^78^ ^]^ Copyright 2019, Wiley‐VCH. b) Constructed chlorin dimers from reduced porphyrin molecules and organized into aggregates with favorable PDT and PTT effects as indicated by PA imaging. Reproduced with permission.^[^ ^81^ ^]^ Copyright 2018, Wiley‐VCH. c) Electrostatic interactions between positively and negatively charged water‐soluble phthalocyanine derivatives resulted in the formation of nanoaggregates with favorable PA imaging and PTT effect. Reproduced with permission.^[^ ^89^ ^]^ Copyright 2019, Wiley‐VCH. d) Gd(III)‐phthalocyanine photosensitizers for dual‐modality imaging‐guided PDT using MRI and PA. Reproduced with permission.^[^ ^91^ ^]^ Copyright 2019, Elsevier Ltd.

The high intrinsic absorbance of the Soret (absorbance peak at 420 nm) and Q bands (multiple absorbance peaks between 500 and 750 nm) is the primary property of porphyrin chromophores.^[^ ^76^ ^]^ Exosomes isolated from tumors were employed as nanocarriers for the photosensitizer Chlorin e6 (Ce6), enabling effective targeted PA imaging in malignancies. Additionally, exosome‐loaded Ce6 demonstrated greater PA signals in imaging than free or liposome‐loaded Ce6.^[^ ^77^ ^]^ Ce6 was grafted onto hyaluronic acid (HA) through redox‐sensitive disulfide bonds in the study by Hu et al. to create amphiphilic polymers for perfluorohexane encapsulation. Ce6 PA intensity could be increased when grafted onto HA, and these nanoprobes demonstrated improved PA imaging in tumors 4 h after injection (**Figure** **4**a).^[^ ^78^ ^]^ Porphyrins may also be combined with other chemicals (e.g., Hf_6_(µ_3_‐O)_4_(µ_3_‐OH)_4_ clusters) to form composite metal–organic frameworks (MOFs) that enable non‐O_2_‐dependent PA imaging‐guided PDT based on the type I mechanism.^[^ ^79^ ^]^ The distance between porphyrins in a covalent framework structure (similar to MOFs structures) prevented self‐quenching, and the lack of metals facilitated biodegradation, which also demonstrated enhanced PA imaging performance.^[^ ^80^ ^]^

Along with the porphyrin attachment to nanocarriers, researchers are focusing on porphyrin chromophore modification. Zheng et al. produced reduced chlorin dimers for PA imaging‐guided synergic PDT and PTT (Figure 4b), making use of reduced porphyrins’ greater extinction coefficient and porphyrin dimers’ self‐assembly in the absence of surfactants.^[^ ^81^ ^]^ Ren et al. synthesized penta‐aza Schiff base expanded porphyrins called texaphyrins, which have a larger core than porphyrins, enabling them to coordinate 1:1 with larger lanthanide metal cations. In comparison of texaphyrin‐Mn, texaphyrin‐Gd, and texaphyrin‐Lu, it was found that texaphyrin‐Mn showed excellent photostability and displayed more effectively in intracellular PA imaging and could avoid toxicity concerns in Gd‐based agents.^[^ ^82^ ^]^

Phthalocyanines are second‐generation photosensitizers that contain four isoindoline substituents and have higher aromaticity than porphyrins, resulting in high absorbance in the Soret and Q bands, with exhibiting particularly high molar extinction coefficients and fluorescence quantum yields in Q band. Unlike porphyrins, phthalocyanines are less phototoxic to skin tissues due to their low absorbance at 400–600 nm.^[^ ^83^ ^]^ In recent years, their increased thermal, chemical, and photostability have garnered more attention. However, phthalocyanine chromophores have the disadvantage of being insoluble in water. To address this disadvantage and enhance nonradiative heat production, a typical technique has been used to produce *π*–*π* stacking compounds between phthalocyanines or phthalocyanines and anthraquinones such as DOX.^[^ ^84^ ^]^ Stabilizers such as amphiphilic polymers,^[^ ^85^ ^]^ liposomes^[^ ^86^ ^]^ are utilized to increase their stability in an aqueous solution. Other ways have been devised in addition to the production of hydrophobic cores using amphiphilic polymers. Wu et al. developed a facile hydrothermal approach for fabricating nanodots on the basis of ZnPc self‐assembly in the presence of citric acid. The produced ZnPc nanodots had a PCE of 54.7% due to internal *π*–*π* stacking and substantial aggregation, which caused fluorescence quenching. ZnPc nanodots demonstrated PA imaging‐guided PTT treatment after surface modification of tumor‐targeting ligands.^[^ ^87^ ^]^ Xie et al. developed a probe containing ZnPc as cores with four PEG arms that could be cut off to reveal hydrophobic ZnPc in the presence of reactive oxygen species (ROS) that was abundant in malignancies, such as H_2_O_2_. Finally, exposed ZnPc grew into large particles in tumors through *π*–*π* stacking, resulting in increased PA signal.^[^ ^88^ ^]^ In comparison, Li et al. synthesized water‐soluble ZnPc compounds by substituting negatively charged 4‐sulfonatophenoxy groups (PcS4) and positively charged 3‐(*N*,*N*,*N*‐trimethylammonium) phenoxy groups (PcN4). These two derivatives were electrostatically assembled into homogenous NPs in an aqueous solution and demonstrated much stronger PA signals than the derivatives alone for PA imaging‐guided PTT (Figure 4c).^[^ ^89^ ^]^ Toriumi et al. investigated the effect of free hydroxyl groups on the NIR absorbance of benzophthalocyanines, and their previous study demonstrated that when hydroxyl groups were present in the benzene ring, an interchange occurred between weakly aromatic (6 phenol) and strongly aromatic (18 quinoline) structures, and the probe was activated in the presence of the target esterase or H_2_O_2_ and gave a strong PA signal at 880 nm.^[^ ^90^ ^]^ Apart from ZnPc, phthalocyanine may also be used to coordinate Gd(III). Zhang et al. developed a unique GdPc probe with a high PA intensity, ^1^O_2_ generation, and a significant T1‐weighted MRI signal that enables the combination of photovacuum and PDT under pulsed laser irradiation (Figure 4d).^[^ ^91^ ^]^ In addition to tumor treatment, PA imaging can also provide imaging information for fat elimination in vivo. The PA agent zinc phthalocyanine tetrasulfonate and the browning agent rosiglitazone were coloaded into hepatitis B core protein complexes and targeting adipose tissue, indicating efficient adipose tissue reduction. PA imaging not only determined the optimal PDT period, but also revealed angiogenesis and morphological alterations associated with the browning of white adipose tissue.^[^ ^92^ ^]^

The conjugated system of naphthalocyanine is larger and the absorbance wavelength is more redshifted than that of porphyrins and phthalocyanines.^[^ ^93^ ^]^ The coordination of Sn(IV) with naphthalocyanine resulted in a redshift of naphthalocyanine's maximum absorbance from 860 to 930 nm. After being modified with a long‐circulating PEG, the probe might be employed as a PA contrast agent for visualizing brain vasculature.^[^ ^94^ ^]^

Porphyrins, phthalocyanines, and naphthalocyanine chromophores, which are often utilized as photosensitizers, have significant in vivo limitations because of their hydrophobicity and the high degree of hypoxia preventing PDT. While the production of nanocarriers and water‐soluble derivatives addresses some of these issues, aggregation‐induced quenching improves nonradiative heat generation, PCE, and PA imaging, but significantly reduces the competitiveness of PDT. As a consequence, obtaining appropriate results for both PA/PTT and PDT is challenging.

##### Boron Dipyrromethene (BODIPY)‐Based Probes

BODIPY is frequently employed as a labeling contrast agent in vitro because of its high fluorescence quantum yield in the visible light spectrum and resistance to fluorescent bleaching. Two ways were often used to move the wavelength of its fluorescence emission or maximum absorption toward the NIR region: 1) modification of BODIPY, such as the addition of a *π*‐conjugation system or heavy atoms, and 2) incorporation of modified BODIPY into liposomes or amphiphilic polymers via hydrophobic interaction and aggregation‐induced quenching, resulting in decreased fluorescence radiation and increased NRET, followed by increased PCE and PA signals in the NIR region.

Merkes et al. synthesized nonfluorescent PyBODIPY by introducing an electron‐rich 1*H*‐pyrrole into the BODIPY chromophore. The electron‐rich 1*H*‐pyrrole could make PyBODIPY's maximum absorption wavelength redshift to 800 nm and resulted in fluorescence emission quenching by photoinduced electron transfer process.^[^ ^95^ ^]^ Gawale et al. extended the *π*‐conjugated system by introducing carbazole groups and heavy atomic iodine into the BODIPY structure, significantly increasing its triplet state quantum yield and ^1^O_2_ generation efficiency, as well as acting as excellent triplet state sensitizers and PA contrast agents.^[^ ^96^ ^]^

**Figure 5:**
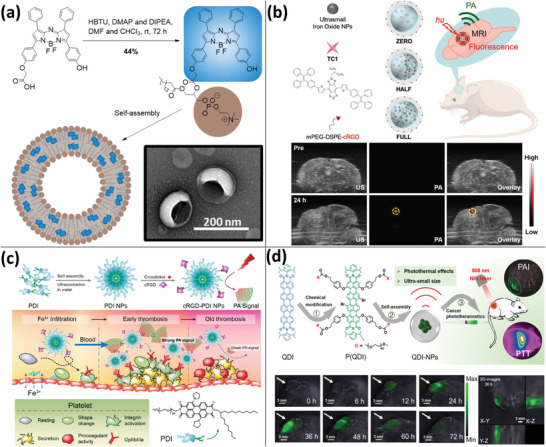
a) Aza‐BODIPY‐lipids generated liposomes containing J‐aggregates in the bilayers, allowing for PA imaging in the NIR range. Reproduced with permission.^[^ ^98^ ^]^ Copyright 2019, Wiley‐VCH. b) PA probes comprised of ultrasmall iron oxide and AIE NPs confined to half of nanospheres, enabling multimodal imaging of orthotopic malignancies in the brain using fluorescence/PA/MRI. Reproduced with permission.^[^ ^100^ ^]^ Copyright 2019, Wiley‐VCH. c) Self‐assembly of amphiphilic perylene‐3,4,9,10‐tetracarboxylic diimide molecules to create PA nanoprobes for early thrombosis detection. Reproduced with permission.^[^ ^102^ ^]^ Copyright 2017, American Chemical Society. d) Self‐assembly of QDI‐grafted PEG nanoaggregates for PA imaging guided‐PTT. Reproduced with permission.^[^ ^103^ ^]^ Copyright 2018, Wiley‐VCH.

Aggregation is another key approach for increasing BODIPY's NIR absorbance. BODIPY's photothermal conversion and PA imaging capability might be increased by hydrophobic contacts between aggregates formed in liposome bilayers or amphiphilic polymer cores.^[^ ^97^ ^]^ Cheng et al. synthesized aza‐BODIPY‐lipid and showed that it could self‐assemble into liposomes, where aza‐BODIPY generated J‐aggregates (dislocation parallel aggregation) between lipid bilayers, and the strong contact between molecular dimers improved the stability of J‐aggregates. The strong interaction between J‐dimers contributed to the stabilization of J‐aggregates and further prevented BODIPYsome vesicles from dissociating above the phase transition temperature, giving it a higher extinction coefficient and quenching efficiency, and allowing it to be used as a PA contrast agent in the NIR region (**Figure** **5**a).^[^ ^98^ ^]^

##### Aggregation‐Induced Emission (AIE) Materials

In contrast to conventional fluorescent dyes that display quenching due to aggregation, AIE materials exhibit modest fluorescence emission in solution but exhibit robust fluorescence emission with substantial Stokes shifts in the aggregated state, garnering extensive interest in bioimaging. By introducing strong electron‐deficient groups (e.g., benzobisthiadiazole, thiophene–thiadiazolobenzotriazole–thiophene) to AIE rotors (e.g., tetraphenyl, triphenylamine) to build electron‐donor and electron‐acceptor (D–A) structures, the maximum absorbance wavelength of AIE materials can be shifted to the NIR region via intramolecular electron transfer. The researchers used this technique to develop and produce AIE‐based PA nanoprobes for in vivo imaging. For instance, Liu's group coprecipitated AIE nanoprobes for NIR‐I PA and NIR‐II fluorescence imaging and effectively realized vasculatures and tumors imaging in mouse brains after RGD modification.^[^ ^99^ ^]^ Additionally, they developed a nanoprobe with iron oxide aggregated on the half side of nanomaterials to prevent the fluorescence quenching and to assure a positive PA effect at 685 nm and MRI signals on orthotopic malignancies in mice brains (Figure 5b).^[^ ^100^ ^]^

##### Others

While perylene diimide (PDI) has garnered much interest for its NIR absorbance and biocompatibility, its low water solubility has hampered its use as a PA contrast agent in vivo. Not only can nanocarriers overcome their low water solubility, but they can also be used to create a *π*–*π* stacking to amplify PA signals. Fan's group has developed a variety of effective PA contrast compounds based on PDI. Perfluorocarbon with a low boiling point was used to load lipid‐soluble small molecules including PDI, photosensitizer, and O_2_, where the amphiphilic PDI molecules acted as stabilizers of the nanodroplets due to their *π*–*π* stacking and long alkyl chains, and vaporization of perfluorocarbon could strengthen PA intensity following temperature increases caused by PDI irradiated by an NIR laser.^[^ ^101^ ^]^ Conjugation of the lipid‐soluble PDI with water‐soluble polymers (e.g., PEG) results in the formation of amphiphilic polymer molecules in which the PDI forms a hydrophobic core through hydrophobic interactions and *π*–*π* stacking. Cui et al. synthesized PDI nanomicelles and modified them with cyclic‐RGD to precisely target early thrombi. Through PA imaging, the nanoprobes were able to efficiently identify normal arteries, and early and late thrombi (Figure 5c).^[^ ^102^ ^]^ While modification of PDI may significantly increase its water solubility, nanomaterials based on coprecipitation or self‐assembly may experience dissociation in the physiological state in vivo, and this dissociation may further result in aggregate formation, limiting their potential.

The core‐expanded quaterrylenediimide (QDI) emits NIR light with a wavelength of 800 nm. However, due to its high nonpolarity and rigid *π*‐system, it has exceptionally low water solubility. To overcome this issue, Yin's group grafted water‐soluble and biocompatible polymers (e.g., PEG or polyacrylic acid) onto the QDI molecule to create amphiphilic polymers that self‐assembled in an aqueous solution to generate NPs less than 10 nm (Figure 5d).^[^ ^103^ ^]^ The aggregates exhibited a more defined monomer structure with a greater PCE and a favorable PA imaging effect, and the reduced size of the NPs facilitated their elimination in vivo.^[^ ^104^ ^]^

#### 2.2.2 Organic PA Nanoprobes

##### SPNs

SPNs have attracted considerable interest in the area of bioimaging due to their exceptional optical characteristics and biocompatibility, owing to their structure including D–A alternatively covalently linked to *π*‐conjugated backbones. Not only can the absorbance of SPNs be shifted from the NIR‐I to the NIR‐II region by tuning the D–A structure, but they can also be enhanced in terms of PCE and PA signal by increasing NRET and reducing fluorescence emission through structural design or enhancing the PA signal via surface modification by reducing heat exchange with the solvent. In comparison to inorganic contrast agents such as AuNRs, CuS, and carbon nanomaterials, SPNs exhibited no long‐term biotoxicity and overcame the limitations of needing nanocarriers wrapping and having poor photostability. We discuss four topics in this section: small molecules with D–A structure and oligomeric semiconductor polymers, SPNs for NIR‐I PA imaging, SPNs for NIR‐II PA imaging, and design strategies of SPNs.

**Figure 6:**
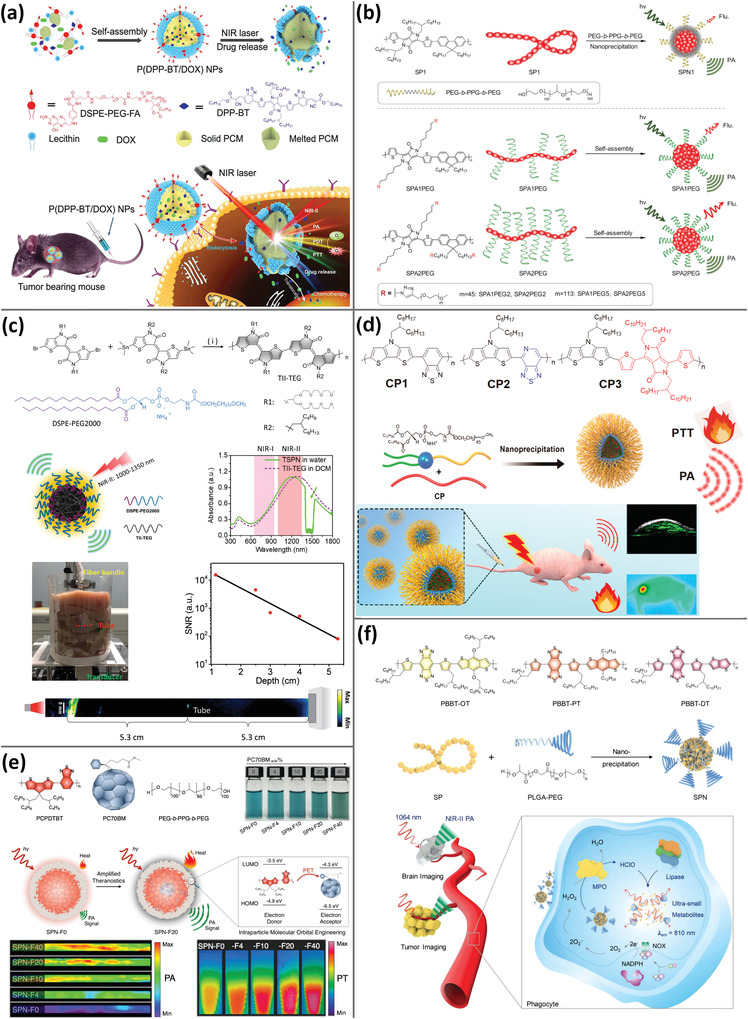
SPNs‐based PA imaging. a) NPs loading small molecules DPP‐BT, DOX, and natural PCM for fluorescence/PA imaging‐guided chemo/PTT/PDT. Reproduced with permission.^[^ ^105^ ^]^ Copyright 2019, Wiley‐VCH. b) The effect of PA imaging on semiconductor amphiphilic polymers with varying degrees of PEG grafting and molecular weights. Reproduced with permission.^[^ ^112^ ^]^ Copyright 2016, Wiley‐VCH. c) SPNs based on thienoisoindigo for NIR‐II PA imaging with centimeter‐depth tissue penetration. Reproduced with permission.^[^ ^120^ ^]^ Copyright 2017, Wiley‐VCH. d) Molecular engineering‐based design of SPNs and investigation of the effect of electron acceptors with varying electron‐absorbing abilities (benzothiadiazole, pyridylthiadiazole, and diketopyrrolopyrrole) on the PA imaging ability of SPNs, demonstrating that SPNs with diketopyrrolopyrrolopyrrole as electron acceptors perform better. Reproduced with permission.^[^ ^124^ ^]^ Copyright 2017, American Chemical Society. e) Molecular orbital engineering was used to develop and prepare PA contrast agents consisting of SPNs and optical dopant fullerenes for PA imaging‐guided PTT. Reproduced with permission.^[^ ^127^ ^]^ Copyright 2016, American Chemical Society. f) After lipase metabolism, 30 nm SPNs were converted to 1 nm ultrasmall metabolites with excellent NIR‐II PA imaging and hepatic and renal excretion capabilities. Reproduced with permission.^[^ ^130^ ^]^ Copyright 2019, Wiley‐VCH.

a) Small molecules with D–A structure and oligomeric semiconductor polymers: In contrast to SPNs, small molecules with D–A structures are also capable of PA imaging and have a well‐defined structure, which enables improved purification and the possibility of molecular design. Wang et al. synthesized diketopyrrolopyrrole‐benzothiadiazole (DPP‐BT) probes for PTT and PA imaging using a two‐step procedure in which electron‐absorbing benzothiadiazole was conjugated to diketopyrrolopyrrole. Wrapping DPP‐BT, DOX, and organic PCM with amphiphilic lecithin for single NIR laser triggered, NIR‐I absorbance, and NIR‐II fluorescence imaging, PA imaging‐guided targeted chemo/PTT were performed following folic acid modification (**Figure** **6**a).^[^ ^105^ ^]^ Ye et al. designed and synthesized BBTD‐1302 with a D–A–D structure, using common benzobisthiadiazole as an electron acceptor and *N*,*N*‐dimethylamino group as an electron donor, which were connected by double thiophene rings and styrene moieties as a bridge, with two thiophene manifesting a transconfiguration and being almost coplanar with the styrene and benzene units, which made it possess a strong absorbance at 942 nm and was used in NIR‐II fluorescence/PA imaging‐guided PTT.^[^ ^106^ ^]^ DSPE‐PEG nanoprecipitation is often used to produce SPNs with dissociation potential. To increase the stability of amphiphilic oligomeric semiconductor polymers, they might be covalently crosslinked using PEG.^[^ ^107^ ^]^

b) NIR‐I SPNs for PA imaging: SPNs having an absorbance in the NIR‐I range (650–950 nm) may be employed as PA imaging probes. Polymer dots based on diketopyrrolopyrrole (electron acceptor)‐dithiophene (electron donor) polymers (DPP‐DT),^[^ ^108^ ^]^ benzodithiophene (electron donor)‐isoindigo derivative (electron acceptor) polymers (BDT‐IID),^[^ ^109^ ^]^ and poly[2,6‐(4,4‐bis‐(2‐ethylhexyl)‐4H‐cyclopenta[2,1‐*b*;3,4‐*b*′]dithiophene)‐*alt*‐4,7(2,1,3‐benzothiadia‐zole)] (PCPDTBT)^[^ ^110^ ^]^ were produced for in vivo tumor PA imaging or tracing of cardiomyocytes obtained from embryonic stem cells.

Other imaging modalities and functions, when paired with PA imaging, may give additional information about the distribution of SPNs in vivo. PCPDTBT, a cyclopentane‐dithiophene‐benzothiadiazole‐based SPNs, was coprecipitated with THF‐dispersed Fe_3_O_4_ and amphiphilic polymers to provide multimodal imaging contrast agents for magnetic particle imaging, MRI, fluorescence/PA imaging (700 nm).^[^ ^111^ ^]^ Pu's group explored the PA characteristics and fluorescence emission of amphiphilic SPNs grafted PEG in further detail. In comparison to nanoprecipitated SPNs, the PEG grafting degree of amphiphilic SPNs had no effect on the PA signal strength at 680 nm. The SPNs with more PEG grafting had a looser interior structure and a reduced concentration of semiconductor fragments, which prevented fluorescence quenching and attenuation caused by aggregation, resulting in greater fluorescence emission (Figure **6**b).^[^ ^112^ ^]^

c) NIR‐II SPNs for PA imaging: The NIR‐II band (1000–1700 nm) fluorescence is more attractive than the NIR‐I region due to less tissue scattering, less autofluorescence, and a better S/N ratio, but a stronger water absorption that may not be disregarded, particularly for imaging brain tumors. Additionally, the maximum permitted exposure intensity in the NIR‐II region is higher (e.g., 1.0 W cm^−2^ for 1064 nm) than it is for the 808 nm (0.33 W cm^−2^).^[^ ^113^ ^]^ The researchers altered the structure of D–A to further lower the transition's energy bandgap and relocated its absorbance to the NIR‐II region. Electron donors such as thiophene and benzodithiophene are often employed, while electron acceptors such as benzodithiadiazole, thiazole‐benzotriazole, and thiophene‐isoindigo are frequently utilized. For PA imaging in the NIR‐II region, a variety of SPNs based on the D–A structure have been created. SPNs were synthesized using an electron‐dense compound ((4,8‐bis((2‐octyldodecyl)oxy)‐benzo[1,2‐*b*:4,5‐*b*′]dithiophene‐2,6‐diyl)bis(trimethylstannane)) as an electron donor and electron deficient (4,8‐bis(5‐bromo‐4‐(2‐ethylhexyl)thiophen‐2‐yl) benzo[1,2‐*c*:4,5‐*c*′]bis[1,2,5]thiadiazole) as the alternately linkages, which was effectively used to enable PA imaging of in situ glioblastoma at 1064 nm with improved accumulation in glioma after the addition of the targeting ligand.^[^ ^114^ ^]^ Li et al. coloaded hydrophobic semiconductor polymers and the NO gas‐generating precursor S‐nitrosothiol in the single nanocarriers using the nanoprecipitation method and used the photothermal effect of SPNs to trigger NO release in order to achieve PA imaging‐guided synergistic treatment following a single laser irradiation.^[^ ^115^ ^]^

The fabrication of D–A1–D–A2 SPNs by adding various electron acceptors into the SPNs is another critical method for narrowing the bandgap and achieving NIR‐II PA imaging. Typically, D–A1–D–A2 SPNs were synthesized by linking electron‐absorbing diketopyrrolopyrrole compounds alternatively with electron‐donating thiophene structures at both ends. In 2017, Pu's group developed SPNs for NIR‐II PA imaging for the first time, and they made stronger electron‐absorbing thiadiazoloquinoxaline groups replaced the electron‐donating thiophene in control group. The D–A1–D–A2 SPNs were effectively used to image the vasculature of the rat brain, and the S/N ratio of NIR‐II imaging was 1.5 times that of NIR‐I imaging.^[^ ^116^ ^]^ Small‐sized or metabolizable SPNs for NIR‐II PA imaging have been generated using nanoprecipitation process with Triton X‐100 or microfluidic techniques.^[^ ^117^, ^118^, ^119^ ^]^ SPNs are often formed by linking D–A monomers together to produce rigid planes with electron‐pulling, delocalized *π*‐bonds and lowering the bandgap in order to achieve NIR‐II absorbance. In reference to thienoisoindigo's use in organic electronics, Wu et al. synthesized thienoisoindigo‐based semiconductor homopolymers with a rigid planar electron deficient structure, a narrow bandgap, and a high absorbance at 1100–1300 nm. They then converted this semiconducting polymer into water‐soluble NPs that could penetrate 5.3 cm thick chicken breast and exhibit PA signal at 1064 nm (Figure 6c).^[^ ^120^ ^]^

d) Design strategies of SPNs: To further increase the PA imaging capacity of SPNs (PA intensity, redshift of the maximum absorbance wavelength to the NIR‐II region, and PCE), electron quenchers or strong electron acceptors may be used to quench fluorescence. Introducing stronger or more electron acceptors may help lower the bandgap energy, as well as improvement of the surface properties of SPNs are acting as effective strategies.^[^ ^121^, ^122^ ^]^ Different electron acceptors were added into SPNs by molecular engineering procedures to examine the correlation between their structure and PA signal. Zhang et al. synthesized three SPNs using thiophene as the electron donor, diketopyrrole as the constant electron acceptor, and thienothiophenen, thienoisoindigo, and benzobisthiadiazole as the second electron acceptors. SPNs containing benzobisthiadiazole as the second electron acceptor demonstrated the greatest absorbance at 1280 nm, the largest PA signals, and the highest PCE due to their distinct electron‐withdrawing and reduced bandgap capacities.^[^ ^123^ ^]^ Liu's group explored the influence of electron acceptor structure on the PA signal of SPNs by molecularly designing the electron acceptor structure in SPNs. Three SPNs with significant intramolecular charge transfer (ICT) were produced using dithiopyrrole as a donor and benzothiadiazole, pyridylthiadiazole, and diketopyrrole as acceptors. Due to the higher electron‐absorbing capacity of diketopyrrole compared to those two, SPNs containing diketopyrrole were able to retain a nearly planar structure and showed increased charge transfer ability, more efficient fluorescence quenching, and a stronger PA signal (Figure 6d).^[^ ^124^ ^]^ Liu et al. investigated the relationship between the chemical structure and the PA property using dithieno[3,2‐*b*:2′,3′‐*d*]pyrrole as a donor and benzochalcogenodiazoles as acceptors (chalcogenide components in the acceptor include oxygen, sulfur, and selenium). The maximum absorbance wavelength was discovered to be redshifted when the chalcogenide components were changed from oxygen to sulfur to selenium. The redshift from oxygen to sulfur happened owing to oxygen's larger electron‐deficient action, while the redshift in selenium occurred due to a reduction in aromaticity. Its maximum absorbance coefficient decreased with the oxygen–sulfur–selenium sequence due to a decrease in electronegativity, which resulted in a decrease in the acceptor unit's electron‐deficiency, which prevented the formation of a stable charge separation state, resulting in the decrease in absorption coefficient.^[^ ^125^ ^]^ In addition to the previously described techniques of reducing the energy bandgap and fluorescence, Zha et al. developed a novel effective approach based on twisted intramolecular charge transfer (TICT) to accelerate NRET. They synthesized novel SPNs using 4,8‐bis((2‐ethylhexyl)oxy)benzo[1,2‐*b*:4,5‐*b*′]dithiophene as an electron donor and [1,2,5]thiadiazolo[3,4‐*g*]quinoxaline as an electron acceptor, in which the alkoxyphenyl, alkylthiophene, and ester functional groups were introduced as substituent groups in the electron acceptors, respectively. It was discovered that the ester‐substituted system exhibited a stronger PA signal, which was attributed to the stronger TICT effect in this system, as well as higher reorganization energy and lower adiabatic energy, resulting in a higher photoinduced nonradiative decay, which resulted in a stronger PA signal.^[^ ^126^ ^]^ By lowering fluorescence radiation and increasing NRET, electron quenchers and strong absorbing electron doping may introduce photoelectrons to increase PA intensity. Pu's group designed and prepared PA imaging nanostructures with binary optical components, and they calculated the molecular orbitals of the primary semiconductor polymer and secondary optical dopant fullerene (PC70BM) using molecular orbital engineering to align their molecular orbitals strictly, which was more favorable for photoinduced electron transfer from the SPNs to the optical dopant, thereby increasing the PA signal intensity and PTT effect (Figure 6e).^[^ ^127^ ^]^ Additionally, their group added strong electron‐absorbing groups, such as benzothiadiazole, into the SPNs to cause fluorescence quenching, which increased NRET and resulted in an increase in PA signal.^[^ ^128^ ^]^

The metabolizability of semiconductor polymers in vivo has also received more attention. One strategy that is often used is to manufacture ultrasmall or biodegradable SPNs. Using the powerfully oxidizing HClO produced in the presence of myeloperoxidase and H_2_O_2_ in immune cells, Pu's group inserted double bonds into SPNs and observed that their absorbance at 819 nm was totally lost after 48 h in RAW264.7 cells after lipopolysaccharide stimulation.^[^ ^129^ ^]^ Following that, they developed three biodegradable SPNs using similar concepts. Along with myeloperoxidase, lipase was added to synergistically degrade SPNs, and they discovered that nonfluorescent SPNs (30 nm) were converted to NIR fluorescent ultrasmall metabolites (≈1 nm) in vivo that exhibited NIR‐II (1064 nm) PA imaging and were excreted via the renal and hepatic systems (Figure 6f).^[^ ^130^ ^]^

Due to their exceptional photostability, chemical inertness, and high PCE, SPNs are one of effective contrast agents for PA imaging in vivo. Their absorbance wavelength and coefficient of absorption may be changed by modifying the structure of the electron donors and acceptors. However, several disadvantages continue to restrict their utilization. First, whereas nanoprecipitation is the primary method for preparing SPNs, noncovalent crosslinking results in leakage, decreased stability, and aggregation upon dissociation in vivo. Second, since SPNs have a large PA signal at the expense of diminished fluorescence emission, they are less capable of balancing fluorescence and PA imaging, and the development of multimodal imaging and theranostic SPNs should be enhanced. Third, although biodegradable SPNs have been examined, more research on degradable SPNs in the tumor or other disease microenvironment should be further investigated.

##### Polymers Derived from Nature

**Figure 7:**
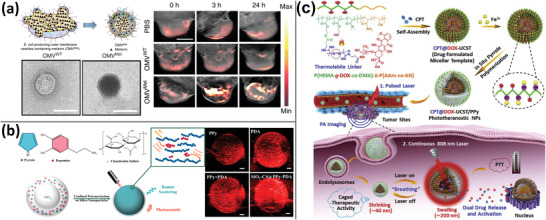
a) Bacterial outer membrane vesicles containing melanin for PA imaging and PTT. Reproduced with permission.^[^ ^131^ ^]^ Copyright 2019, Springer Nature. b) PDA‐doped PPy as intrinsic Raman and PA imaging dual‐mode semiconductor polymers synthesized by a one‐pot approach to improve their PA imaging capacity via intermolecular energy transfer. Reproduced with permission.^[^ ^133^ ^]^ Copyright 2018, American Chemical Society. c) PPy‐loading CPT, DOX to increase their upper critical solubility temperature for PTT and PA imaging‐guided chemotherapy. Reproduced with permission.^[^ ^135^ ^]^ Copyright 2018, Elsevier Ltd.

Melanin is a naturally occurring pigment that is often employed in melanoma and pigmented lesions as an endogenous PA imaging contrast agent. Artificial melanin NPs are typically generated in two ways: dispersion of eumelanin NPs in water and oxidative polymerization of dopamine (DA) to fabricate PDA NPs. Gujrati et al. created bacterial outer membrane vesicles containing melanin using bioengineered *Escherichia coli*, which displayed a higher tumor PTT effect and PA signal than wild‐type vesicles (**Figure** **7**a).^[^ ^131^ ^]^ In comparison to melanin NPs, oxidative polymerization of DA is more suitable for integrating various functional probes on the surface and may produce NPs with a variety of morphologies, including solid NPs, mesoporous structures, and nanocapsules.^[^ ^132^ ^]^ Lin et al. synthesized PDA‐doped polypyrrole (PPy) as intrinsic Raman and PA imaging dual‐mode semiconductor polymers using SiO_2_ as templates. PDA and PPy were doped physically owing to their distinct polymerization mechanisms and conducting electrons may be transported between PPy and PDA. As a consequence of intermolecular energy transfer, their PA intensity may be increased, and the NIR absorbance caused by the resonance Raman effect resulted in an increase in its Raman scattering intensity as well (Figure 7b).^[^ ^133^ ^]^ PDA's rich amino and catechol groups allow for easy modification with other chemicals. PDA NPs have coordination sites for metal ions due to the catechol structure. Lemaster et al. also discovered that doping metal ions into PDA NPs can increase their PA signal intensity, with the T1‐weight MRI contrast agent Gd(III)‐doped PDA NPs exhibiting the highest PA signal intensity due to the catechol group's coordination with the metal ion improving the absorbance cross‐section.^[^ ^134^ ^]^

Although biocompatible PDA NPs are simple to synthesize, their degradation time in vivo is quite long, their PCE is low, the ligand stability of the catechol structure with metal ions is easily affected by pH, and they exhibit quenching of fluorescence from coloaded fluorescent probes, all of which are unfavorable factors limiting the applications of PDA‐based nanomaterials in vivo.

##### Organic Conductive Conjugated Polymers

PPy and polyaniline are organic conductive conjugated polymers, which both exhibit excellent photothermal stability and biocompatibility. Yang et al. next employed PPy as a photothermal material to fabricate a photothermally triggered release nanocarrier encapsulating two chemotherapeutic drugs (camptothecin (CPT) and DOX prodrug) for use in combination with PTT under PA imaging‐guided chemotherapy (Figure 7c).^[^ ^135^ ^]^ Zhang et al. used a one‐pot approach to encapsulate polyaniline around bis(2‐ethylhexyl)sulfosuccinate vesicles for pH‐sensitive NIR‐II (1064 nm) PTT and PA imaging (970 nm).^[^ ^136^ ^]^ However, both PPy and polyaniline have drawbacks, including difficult surface modification. Additionally, polyaniline alone is only stable at very low pH values, while PPy has limited water solubility.

## 3 “Turn‐On” PA Imaging Probes

Through passive or active accumulation, “always‐on” PA contrast agents provide a stronger PA signal in pathological tissue than in normal tissue. Due to the fact that normal tissues (particularly liver) are also perfused with a certain concentration of contrast agents, the S/N ratio and specificity of these PA contrast agents are insufficient. Liu et al. designed a reversible photoswitching nanoprobe to decrease the background signal in PA imaging. They used a common photoswitchable molecule (dithienylethene‐containing *β*‐diketone) that could be converted to a closed form with considerable absorbance in the NIR region and an open form without absorbance in the NIR region when exposed to UV and red light, respectively. It was also mixed with upconversion nanomaterials whose surface was modified with amphiphilic polymers. Upconversion nanomaterials could generate UV light when irritated with a 980 nm laser, prompting structural alteration, and amplifying their PA signal. A 680 nm laser might be used to disrupt the PA signal. This probe significantly removed the background signal, and it (0.5 nm) was successfully deployed in hemoglobin solution, enhancing the S/N ratio and imaging sensitivity.^[^ ^137^ ^]^

Only at lesion locations may activatable biomaterials be triggered by disease‐related small molecules or disease microenvironment.^[^ ^138^, ^139^, ^140^ ^]^ The creation of activatable imaging probes can efficiently minimize background signal and increase imaging sensitivity and specificity, which is particularly crucial for the identification of minute lesions. Dynamically switchable MRI contrast agents, for example, have been developed. Alteration in their structures may be driven by disease microenvironment, resulting in a “T2 to T1” switching or dual enhancement of “T1 and T2” signals to increase MRI accuracy.^[^ ^141^ ^]^ To increase the specificity and sensitivity of the PA contrast agents, researchers adopted a similar strategy to design a series of “turn‐on” PA contrast agents that undergo structural changes in the presence of certain molecules, altering their absorbance spectra and PA signal strength. They were categorized in this part as “single‐wavelength detection” and “ratiometric detection,” with “ratiometric detection” further classified and summarized as “internal reference detection” and “seesaw detection.” **Table** **2** summarizes the “single‐wavelength detection” PA probes.

**Table 2:** “Single‐wavelength detection” PA probes in physiological imaging

Materials	Detecting substances	Responsive parts	Detective wavelength	Detection range	Detection limit	Applications	Refs.
HCy‐Cit‐Val and HCy‐Gly‐Leu‐PheGly	Cathepsin B	ICT between hemicyanine and Val‐Cit or Gly‐Phe‐Leu‐Gly	675 nm	0–30 U L^−1^	HCy‐Cit‐Val or HCy‐Gly‐Leu‐PheGly was 0.407 or 0.723 U L^−1^	Cathepsin B activation	[142]
LET‐3	Alkaline phosphatase	Phosphate moiety	710 nm	0.00–2.00 U mL^−1^	0.8 U mL^−1^	Endogenous alkaline phosphatase detection	[146]
Au‐H1/H2 PA nanoprobes	MUC1‐specific sialic acid	Proximity‐induced hybridization chain reaction triggered gold nanoassemblies formation	680 nm	0–80 nmol L^−1^	10 nmol L^−1^	Mucin 1 (MUC1)‐specific glycosylation	[147]
MNP‐PANI	pH	Polyaniline	800 nm	pH 2.0–6.0	Not mentioned	Gastric acid secretion pH measurement	[151]
CySO_3_CF_3_	Peroxynitrite	Trifluoromethyl ketone moiety, an ONOO^−^‐responsive unit	686 nm	0–10 µmol L^−1^	145 nmol L^−1^	Tumor imaging	[156]
AuNCs@SiO_2_	H_2_O_2_	Aggregation	1280 nm	0–100 µmol L^−1^	Not mentioned	Tumor imaging	[161]
MnMoO*_X_* Nanorods	GSH	Mo(VI)	830 nm	0.5–10 mmol L^−1^	0.5 mmol L^−1^	Tumor imaging	[162]
PACD*x*	GSH	Gemcitabine prodrugs	690 nm	0–10 mmol L^−1^	0.39 mmol L^−1^	Elevated glutathione in lung cancer for companion diagnostic applications	[163]
LET‐2	Cu^2+^	Dipicolylethylenediamine	715 nm	0–20 µmol L^−1^	10.8 × 10^−9^ mol L^−1^	Cu^2+^ detection	[166]
RPS1	Cu^2+^	RPS1	710 nm	0–35 µmol L^−1^	90.9 nmol L^−1^	Visualization of copper(II) in mice with Alzheimer's disease	[167]
MTR‐CO	CO	A Tsuji–Trost reaction in presence of Pd^2+^	690 nm	0–60 µmol L^−1^	0.66 µmol L^−1^	Imaging of endogenous carbon monoxide in the murine inflammation model	[170]
Cy‐N	NADPH	Double bond rearrangement of quinolinium moiety	720 nm	0–70 µmol L^−1^	Not mentioned	Tumor imaging	[171]
Methylene blue	Heparin	Methylene blue	680 nm	0–6.4 U mL^−1^	14.2 mU mL^−1^	Clotting time and therapeutic drug monitoring of heparin	[174]
SiRho‐HD	ONOO^−^	SiRho‐HD	715 nm	0–30 µmol L^−1^	1.3 µmol L^−1^	Imaging of peroxynitrite in drug‐induced acute kidney injury	[209]
Au‐MUA5‐TMA5	pH	Aggregation	808 nm	pH 6.0–7.0	ΔpH 0.7	Tumor imaging and PTT	[210]

### 3.1 Single‐Wavelength Detection

“Single‐wavelength detection” PA nanoprobes were identified in this review as responsive PA nanoprobes only with one peak altering when encountering detective chemicals. These responsive PA nanoprobes were previously developed for the detection of disease‐related small biomolecules, enzymes, and other chemicals via PA imaging. Core–shell nanostructures, nanoaggregation triggered by microenvironment, and introducing responsive groups in “always‐on” small organic molecule PA probes have been investigated for “single‐wavelength detection” PA nanoprobes.

#### 3.1.1 Detection of Enzymes

The expression of certain enzymes was shown to be closely associated to illness. The invention of enzyme‐responsive PA probes can provide dynamic, noninvasive measurement of enzyme activity in vivo, allowing for disease progression monitoring.

##### Cyanine and Hemicyanine‐Based Small Organic Molecules for Detection of Marker Enzymes

**Figure 8:**
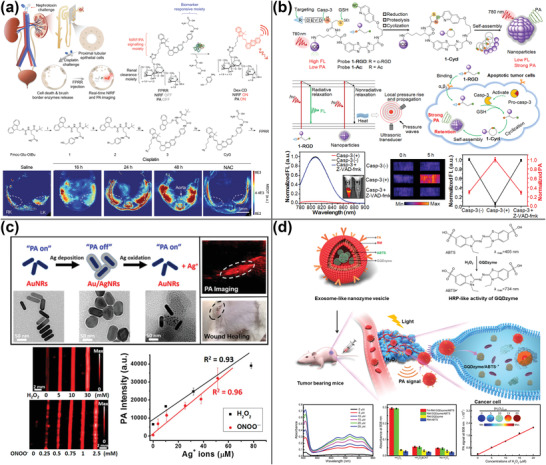
Signal wavelength responsive PA imaging probes. a) A *γ*‐glutamyltransferase‐responsive PA probe was constructed using dextran, semicarbazone, and *γ*‐glutamate, in which the amide bond adjacent to *γ*‐glutamate was specifically cleaved in the presence of *γ*‐glutamyltransferase, allowing for the diagnosis of acute kidney injury using NIR fluorescence and PA imaging. Reproduced with permission.^[^ ^144^ ^]^ Copyright 2020, Wiley‐VCH. b) Caspase‐3 activated PA probes based on peptide substrate cleavage and biocompatible macrocyclization‐mediated self‐assembly for detecting caspase‐3 activity in PA imaging. Reproduced with permission.^[^ ^149^ ^]^ Copyright 2018, Wiley‐VCH. c) AuNRs with Ag masking shells etched with H_2_O_2_ or ONOO^−^ were employed in anti‐infection treatment and monitored by PA imaging. Reproduced with permission.^[^ ^154^ ^]^ Copyright 2018, American Chemical Society. d) Nanoenzymes composed of erythrocyte membrane‐encapsulated graphene QDs and their substrate ABTS were produced to enable responsive imaging of H_2_O_2_. Reproduced with permission.^[^ ^158^ ^]^ Copyright 2019, American Chemical Society.

Cyanine and hemicyanine are the most extensively used “always‐on” PA probes. Researchers have created cyanine or hemicyanine‐based PA probes for the detection of metal ions, marker enzymes, ROS radicals, and other molecules based on ICT. Three components make up cyanine or hemicyanine dyes used to identify marker enzymes and physiological small molecules: 1) a chromophore (cyanine or hemicyanine dye scaffolds), 2) a responsive group activated by the tested compounds (for example, enzyme substrate, ROS‐cleavable parts or metal ions ligand) and capable of masking the probe's PA signal, and 3) a functional group other than PA imaging, such as targeting ligands. In the case of marker enzyme detection, the substrate could be removed in presence of marker enzyme and then their PA signals could be recovered. On the basis of the idea of preventing intramolecular electron transport, Chen et al. developed two cyanine probes for detecting Cathepsin B activity in malignancies. They connected two Cathepsin B substrate structures (Cit‐Val and Gly‐Leu‐Phe‐Gly) to hemicyanine, which was selectively eliminated by Cathepsin B, leading to the recovery of fluorescence emission at 705 nm and PA signal at 675 nm, allowing for the detection of Cathepsin B in tumors.^[^ ^142^ ^]^ To enhance renal metabolism or prolong blood circulation time in vivo, Pu's group developed and synthesized a variety of semicarbocyanine dye‐based probes.^[^ ^143^, ^144^, ^145^ ^]^ The dextran fraction was coupled to the semicarbocyanine dye, and the PA signal of these probes might be lighten up by the substrate fraction in the presence of ROS or marker enzymes. Based on urokinase‐type plasminogen activator (uPA) expression in invasive breast cancer, Li et al. designed a responsive probe with four components (a kidney‐cleavable dextran backbone, a self‐immolative linker, the NIR dye CyN3OH, and a uPA‐cleavable substrate) that could be activated to produce specific fluorescence and PA signals upon contact with breast cancer tissues with high uPA expression, and was able to effectively differentiate infiltrating and noninfiltrating breast tumor tissues.^[^ ^143^ ^]^ Cheng et al. developed a PA detection probe composed of dextran, hemicyanine, and *γ*‐glutamate for the detection of *γ*‐glutamyl transferase, which was overexpressed in early acute kidney injury. In the presence of *γ*‐glutamyl transferase, the amide bond next to *γ*‐glutamate might be preferentially cleaved, effectively increasing NIR fluorescence and PA signals (**Figure** **8**a).^[^ ^144^ ^]^ Gao et al. synthesized a “turn‐on” NIR fluorescence and PA dual‐mode responsive probe by adding phosphate to semicarbazide and specifically cleaving the phosphate in the structure in the presence of alkaline phosphatase, where the hydroxyl enhanced the electron‐giving capability and resulted in an enhanced PA signal at 710 nm.^[^ ^146^ ^]^

##### Nanoaggregation Stimulated by Marker Enzymes

The formation of aggregates induced by the tumor microenvironment is also a critical strategy for “turn‐on” PA imaging, where marker enzymes are intended as aggregation‐triggered response components in malignancies. Typically, such probes begin as small probe precursors that may operate as enzyme‐catalyzed substrates or aggregate in the presence of small molecules such as glutathione (GSH). Smaller precursors facilitate tumor tissue diffusion, while larger aggregates favorably result in tumor tissue retention. Liu et al. then employed a proximity‐induced hybridization chain reaction to aggregate gold NPs and subsequently amplified the PA signal in vivo for glycosylation detection. The particular response between two complementary sequences of glycan probes and protein probes, resulted in aggregated gold NPs in this system, and the PA signal was amplified at 680 nm to provide in situ monitoring of MUC1‐specific sialic acid.^[^ ^147^ ^]^ Wu et al. then developed the IR775‐conjugated Phe‐Phe‐Tyr(H_2_PO_3_)‐OH probe, which could be triggered by tumor‐derived alkaline phosphatase. When interacting with alkaline phosphatase, the phosphate groups were excised, increasing hydrophobicity and aggregation, resulting in self‐quenching of NIR fluorescence but an increase in PA signal.^[^ ^148^ ^]^ Caspase‐3 is activated during the early stages of tumor tissue apoptosis. Wang et al. developed a PA probe based on peptide substrate cleavage and biocompatible macrocyclization‐mediated self‐assembly that can occur only in the presence of caspase‐3, resulting in PA imaging signal enhancement and retention in apoptotic tumor cells. By imaging caspase‐3 activity in vivo with PA imaging, this probe might be used to selectively detect caspase‐3 activity (Figure 8b).^[^ ^149^ ^]^

##### Other Strategies Designed for Detection of Marker Enzymes

Ouyang et al. constructed AIE nanoprobes with a D–A structure, in which the electron donor dihydroxanthene was combined with the hydrophilic electron acceptor quinoline, and dihydroxanthene was further grafted with the nitroreductase substrate nitrobenzyloxydiphenylamino, which quenched fluorescence. Once in contact with nitroreductase, the substrate group was removed and the structure activated to a D–A structure that also displayed high NIR‐II region emission and PA signal, allowing for sequential metastatic imaging from lymph node to lung in mice.^[^ ^150^ ^]^

#### 3.1.2 Detection of pH

The PA signal of polyaniline could alter along with pH value. At pH 4, polyaniline can be converted from the emerald base to the emerald salt state with a high NIR absorbance. Taking advantage of this property, Li et al. wrapped a polyaniline layer on the surface of magnetic nanomaterials and measured the change of PA signal of polyaniline in different pH values for the pH detection of gastric acid.^[^ ^151^ ^]^ However, the conversion pH was lower than the pH of tumor tissue. To address this issue, Tian et al. matched the conversion pH of BSA to that of tumor tissue through an intermolecular acid–base interaction between the carboxyl group of BSA and the imine of polyaniline. This resulted in an increase in PA signal and PTT in the tumor acidic microenvironment.^[^ ^152^ ^]^ In addition to polyaniline pH‐responsive PA imaging characteristics, small molecule probes with pH‐sensitivity are also loaded onto NPs to achieve pH‐responsive PA imaging of tumor tissue. Gao et al. synthesized a pH‐responsive croconium dye with carboxyl groups that were then covalently bonded to PEG to form amphiphilic Croc‐PEG5K. Croc‐PEG5K was then covalently attached to the surface of erythrocyte membrane vesicles. After 780 nm light excitation, the probe generates a pH‐responsive PA signal, enabling pH‐responsive PA imaging and PTT.^[^ ^153^ ^]^

#### 3.1.3 Detection of ROS

ROS has been shown to have a role in a variety of physiological processes and diseases, including apoptosis and inflammation. As a result, ROS detection is essential in oxidative stress‐related diseases. ROS‐detective PA probes based on core–shell nanostructures were constructed, with the shells shielding the cores’ PA signals. Once the target molecules arrive, the surface masking shells may be degraded to retrieve the cores’ PA signal. For example, masking Ag shells covered on typical “always‐on” PA contrast agents AuNRs and the PA signals of AuNRs recovered along with masking Ag shells etching. Kim et al. created composite nanomaterials by enveloping Ag shells over the surface of NIR‐absorbing AuNRs. Masking Ag shells might be etched in the presence of oxidizing radicals such as H_2_O_2_ or ONOO^−^, revealing the PA signals of AuNRs together with the release of Ag^+^. This procedure might be used to monitor the treatment of Gram‐positive methicillin‐resistant *Staphylococcus aureus*‐infected mice wounds dynamically using PA imaging (Figure 8c).^[^ ^154^ ^]^ Mei et al. prepared miniature Au/Ag nanorods for use in MRSA anti‐infection therapy. Ag shells could be etched in K_3_[Fe(CN)_6_] solution allowing the recovery of the PA signal and photothermal effect of AuNRs for activatable NIR‐II PA imaging and photochemical synergistic therapy.^[^ ^155^ ^]^

**Figure 9:**
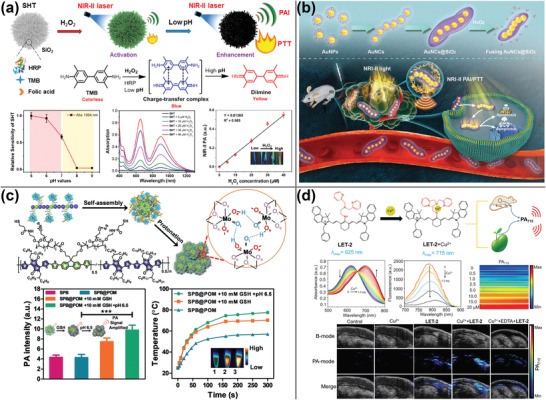
Signal wavelength responsive PA imaging probes. a) HRP and its substrate TMB were loaded in SiO_2_ to accomplish dual responsive PA imaging of H_2_O_2_ and pH with decreased background signal. Reproduced with permission.^[^ ^159^ ^]^ Copyright 2019, American Chemical Society. b) A chain comprising gold NPs was produced with an increased NIR‐II PA signal in the presence of H_2_O_2_. Reproduced with permission.^[^ ^161^ ^]^ Copyright 2020, Wiley‐VCH. c) POM and semiconductor polymer complexed NPs were generated for tumor microenvironment‐responsive PA imaging by POM aggregation in an acidic environment. Reproduced with permission.^[^ ^165^ ^]^ Copyright 2018, Wiley‐VCH. d) The ligand was coupled to the cyclohexene in the IR823 structure to form a PA probe for the detection of Cu^2+^ in bean sprouts and mice. Reproduced with permission.^[^ ^166^ ^]^ Copyright 2019, Wiley‐VCH.

ROS detective PA probed based on cyanine and hemicyanine‐based small organic molecules were also developed. Zhang et al. connected a trifluoromethyl ketone moiety to a sulfonated semicarbonyl dye through the ONOO^−^‐cleavable trifluoromethyl ketone moiety. When ONOO^−^ was present, the ONOO^−^‐induced cascade oxidation‐elimination processes illuminated the probe's NIR fluorescence/PA signal, allowing for in vivo detection of ONOO^−^.^[^ ^156^ ^]^ Chen et al. designed and prepared a probe TPP‐HCy‐BOH specifically responsive to H_2_O_2_ based on ICT principle, which both mitochondria‐targeted TPP moiety and H_2_O_2_‐responsive moiety boronic acid (BOH) were introduced into hemicyanine. Its fluorescence and PA signals were turned off due to the caged hydroxyl group of HCy with inhibited ICT; when targeting into the mitochondria of inflammatory cells, BOH fraction could be removed by excess H_2_O_2_, and its fluorescence and PA signals restored.^[^ ^157^ ^]^ The signal amplification for H_2_O_2_‐detective PA imaging in tumors may be accomplished by combining a peroxidase‐active component with an H_2_O_2_‐responsive probe in the same nanocarrier. Ding et al. used a folate‐modified erythrocyte membrane as a nanocarrier to load graphene quantum dots (QDs) as nanoenzymes with peroxidase activity and a responsive molecule, 2,2′‐azidobis(3‐ethylbenzothiazoline‐6 sulfonic acid) (ABTS), and found that the presence of the nanoenzyme effectively triggered ABTS oxidation (Figure 8d).^[^ ^158^ ^]^ Wang et al. coloaded 3,3′,5,5′‐tetramethylbenzidine (TMB) (with broadband absorption after its oxidation) and horseradish peroxidase (HRP) into mesoporous SiO_2_ NPs. When the substrates reached the tumors, they were oxidized with abundant H_2_O_2_ catalyzed by HRP, and the PA signal was amplified to achieve dual responsiveness to H_2_O_2_ and pH, while simultaneously decreasing the background signal (**Figure** **9**a).^[^ ^159^ ^]^

ROS‐degradable polyphosphonitrile polymer‐loaded gold NPs nanoaggregates allowed targeted imaging of tumor tissues through a reduced PA signal.^[^ ^160^ ^]^ Zhou et al. developed PA nanoprobes for the detection of H_2_O_2_, which consisted of a citric acid‐wrapping chain containing gold NPs and SiO_2_ as a stabilizer. Citric acid was removed off the surface of gold NPs in the presence of H_2_O_2_ to cause higher NIR‐II PA signals (Figure 9b).^[^ ^161^ ^]^

#### 3.1.4 Detection of GSH

GSH was shown to be important in altering the oxidation–reduction status in the physiological state. Gong et al. created MnMoO*_X_* nanorods to monitor GSH, which converted Mo(VI) to Mo(V), converting nanorods to NPs and enhancing PA signal for tumor‐specific imaging.^[^ ^162^ ^]^ Lucero et al. carefully coordinated and adjusted the response of S_N_Ar to create and produce a PA imaging‐based probe for companion diagnostics using gemcitabine prodrugs. This sensor is capable of discriminating between aberrant GSH concentrations (0.1–100 mmol L^−1^) shown in lung cancer models and normal tissues. The authors added hydroxymethyl to the probes to serve as an additional linkage to the gemcitabine when the generated phenol intermediate in the presence of GSH could release the drugs and dyes via 1,4‐elimination, allowing the PA signal to be recovered at 690 nm and achieving tumor site‐specific chemotherapy.^[^ ^163^ ^]^ Due to their adaptable electronic structure, molybdenum‐based polymetallic oxides may enhance NIR absorbance, and molybdenum‐based polymetallic oxides can form larger aggregates through hydrogen bonding in tumor tissues. Meanwhile, GSH found in tumor tissue has the ability to reduce Mo(VI) to Mo(V), and the electron relaxation polarization generated by the electron transfer offers them NIR absorbance. Ni et al. designed ultrasmall size, high oxidation state polyoxometalate (POM) clusters that were acidified and reduced to large size POM aggregates with a high NIR absorbance in the tumor tissue‐specific low pH and high GSH microenvironment, and the small size POM clusters were excreted via the kidney. Increased PA signal was used to accomplish photothermal ablation of 4T1 tumors in mice.^[^ ^164^ ^]^ Yang et al. then compounded POM with semiconductor polymers to increase its ability to accumulate in tumors and found that POM aggregation in an acid microenvironment could further trigger compound aggregation, thereby increasing PA signal intensity and PTT effect via an aggregation‐triggered method (Figure 9c).^[^ ^165^ ^]^

#### 3.1.5 Detection of Metal Ions

The conventional technique for metal ion detection is to incorporate ligands with nitrogen atoms into their structures, where the nitrogen atoms’ lone pair electrons may delocalize to chromophore fractions, hence reducing the delocalization impact when the metal ion is ligated. Zeng et al. detected Cu^2+^ by grafting the chelator dipicolylethylenediamine onto the heptamethine cyanine IR823 analogue. After coordination with Cu^2+^, the electron density of the ligands reduced, reducing the electron‐giving capacity of the amine in the IR823 counterpart, which resulted in its absorbance peak redshifted from 625 to 715 nm and effectively detecting Cu^2+^ in bean sprouts and mice (Figure 9d).^[^ ^166^ ^]^ Along with “turn‐on” PA probes based on cyanine and hemicyanine dyes, analogous small molecules have been shown to operate as internal “turn‐on” PA contrast agents. Wang et al. designed Cu^2+^ detection PA probes based on Alzheimer's medications and the aniline free radical structure. They did so by using electron‐giving groups such as *N*,*N*‐dimethylaniline, which increased the stability of PA in an aqueous solution. Additionally, the probe's relative molecular mass was less than 600, allowing it to penetrate across the blood‐brain barrier and detect Cu^2+^ in brain tissue.^[^ ^167^ ^]^ Mishra et al. conjugated IR780 with Ca^2+^ ligands. When ligated with Ca^2+^, the ligand's electron mobility was blocked (the delocalization of the N atom's lone pair electrons on the ligand), resulting in a smaller absorbance peak and hence a reduced PA signal at 765 nm.^[^ ^168^ ^]^

#### 3.1.6 Other Detection Targets

The “single‐wavelength detection” PA probes for other small molecules detection closely related to physiological and pathological process were also developed. Lucero et al. developed a NO‐responsive PA probe and successfully detected tumor‐derived NO in mice using a two‐phase tuning approach. First, analyzing to identify highly reactive and selective phenylamino triggers that react with NO via *N*‐nitrosylation chemistry; then, screening the NIR‐II platform with a less aggregation‐prone chemical structure.^[^ ^169^ ^]^ Li et al. added allyl formate groups to the anthocyanine structure with NIR absorbance to inhibit intramolecular electron transport, resulting in the reduction of Pd^2+^ to Pd^0^ in the presence of CO. Additionally, the Tsuji–Trost reaction precisely cleaves the allyl formate structure and recovers the PA signal at 690 nm, allowing CO detection in inflammatory tissues in vivo.^[^ ^170^ ^]^ In addition to the foregoing ways for designing activated probes, Tian et al. produced a bimodal NIR fluorescence and PA probe that was sensitive to NAD(P)H through structural modifications in the *π*‐conjugation system. Under normal physiological circumstances, cyanine dyes displayed no fluorescent signal in the NIR region; nevertheless, when they interacted with NAD(P)H, their large *π*‐conjugation structure was displaced, resulting in fluorescence and PA signals appearing.^[^ ^171^ ^]^

Other detections related to the microenvironment were also developed. Cui et al. developed a thermally sensitive semiconductor polymer with a low critical solution temperature of 48 °C. Phase separation occurred beyond this temperature, resulting in aggregation and amplification of the PA signal, hence increasing the tumor S/N ratios.^[^ ^172^ ^]^ Hypoxia is a hallmark characteristic of malignancies; therefore, quantifying tumor hypoxia is critical. Knox et al. synthesized HyP‐1 with an asymmetric structure that has a hypoxia response trigger on one side and a methoxy substituent on the other, employing a highly absorbing and photostable aza‐BODIPY dye as the core structure. In a hypoxic environment, HyP‐1 is capable of binding to heme proteins CYP450 and reducing straight to aniline structure, resulting in a strong S/N ratio of PA signal that is not reliant on the redox cycling.^[^ ^173^ ^]^ Wang et al. discovered a considerable increase in the PA signal of methylene blue following binding to the anticoagulant heparin using the FDA‐approved methylene blue as a contrast agent. Not only is this technique effective for low‐molecular‐weight heparin, but it also retains a high degree of responsiveness in whole blood. For the first time, it was shown that PA imaging could be used to monitor anticoagulant treatment in real‐time.^[^ ^174^ ^]^

Lyu et al. designed and prepared a reaction‐based semiconducting polymer nanoprobes (RSPNs) for protein sulfenic acid detection. They wrapped inert silica and PEG layers on the surface of SPNs and modified sulfenic acid reactive groups on their surface by click chemistry to achieve a specific recognition reaction between RSPNs and protein sulfenic acid. A correlation between the PA signal intensity of this probe and the degree of protein sulfenic acid‐induced response enables the detection of protein sulfenic acid at the cellular level and tumor tissue in vivo.^[^ ^175^ ^]^ Early detection of nonalcoholic fatty liver disease is critical since alterations in the peroxisome's viscosity are strongly associated with this illness. Zhou et al. developed a viscosity‐responsive PA probe composed of a malononitrile rotor structure, a peroxisome‐targeting polypeptide, and a fluorescent thiocyanate dye. When the viscosity was low, the rotor rotated freely and the fluorescence signal was quenched; however, as the viscosity increased, the rotor rotated more slowly, reducing the possibility of nonradiative signals, and both the fluorescence and PA signals increased simultaneously, enabling NIR fluorescence/PA dual‐mode imaging of liver peroxisome viscosity in a mouse model.^[^ ^176^ ^]^

### 3.2 Ratiometric Detection

There are two primary ways of ratiometric PA detection. 1) “Internal reference detection,” in which a sensitive probe is coloaded with an inert probe in the same nanocarrier, and the responsive probe's wavelengths vary while the inert probe's wavelengths remain constant. 2) “Seesaw detection,” a small molecule probe or a nanoprobe coloaded with two contrast agents, in which the PA signal varies in response to irradiation with two distinct wavelengths when various concentrations of target molecules are measured. **Table** **3** summarizes the ratiometric PA probes.

**Table 3:** “Ratiometric detection” probes in physiological imaging

Materials	Detecting substances	Detection types	Responsive parts	Ratio wavelengths	Detection range	Detection limit	Applications	Refs.
NRh‐IR‐NMs	Cu^2+^	Internal reference	Selective Cu^2+^‐responsive probe (NRh)	PA716 (increased)/PA834 (fixed)	0.5–10.0 eq	Not mentioned	Deep tissue detection of Cu^2+^ in living organisms	[177]
CuS@PB	ONOO^−^	Internal reference	Prussian Blue	PA970 (fixed)/PA710 (decreased)	2–25 µmol L^−1^	838 nmol L^−1^	ONOO^−^ imaging in drug‐induced hepatotoxicity	[178]
DATN	NO	Internal reference	NRM	PA680 (increased)/PA950 (fixed)	0–30 µmol L^−1^	Not mentioned	Tumor imaging	[179]
SOA‐based PA nanoprobe	ClO^−^	Internal reference	SOA/NIR775	PA780 (fixed)/PA680 (decreased)	0–12 µmol L^−1^	1.3 µmol L^−1^	Tumor imaging	[186]
RSPN	O_2_^•−^	Internal reference	O_2_^•−^ responsive molecule/OIM	PA690 (increased)/PA800 (fixed)	0–150 µmol L^−1^	Not mentioned	Determination of O_2_^•−^ within aortic atherosclerosis	[187]
OSN‐B1	ONOO^−^	Internal reference	OSN	PA750 (increased)/PA680 (fixed)	0–10 µmol L^−1^	100 × 10^−9^ mol L^−1^	Tumor imaging	[188]
CR‐POM nanoprobe	GSH	Seesaw	Croconaine dye (700 nm) and POM (866 nm)	PA866 (increased)/PA700 (decreased)	0–14 mmol L^−1^	0.512 mmol L^−1^	Simultaneous accurate quantification of GSH levels	[189]
APSel	Selenol	Seesaw	Bis(2‐hydroxyethyl)disulfide	PA690 (increased)/PA860 (decreased)	0–5 µmol L^−1^	73 nmol L^−1^	Selenol imaging in autoimmune hepatitis	[190]
IR806‐PDA	GSH	Seesaw	IR806‐PDA	PA820 (increased)/PA680 (decreased)	0–2000 µmol L^−1^	3.13 µmol L^−1^	Tumor imaging	[191]
LPhCy7	MeHg^+^	Seesaw	hCy7	PA860 (increased)/PA690 (decreased)	0–5 µmol L^−1^	2.0 ppb	MeHg^+^ detection	[192]
OEG‐Aza‐BODIPY‐BAPE	H_2_O_2_	Seesaw	Aza‐BODIPY backbone attached with benzeneboronic acid pinacol ester moiety	PA825 (increased)/PA720 (decreased)	0–100 µmol L^−1^	0.6 µmol L^−1^	Hydrogen peroxide detection	[194]
BDP‐DOH	O_2_^•−^ and GSH	Seesaw	BDP‐DOH	PA750 (increased)/PA680 (decreased)	0–4 µmol L^−1^ (for O_2_^•−^)	0.03 µmol L^−1^ (for O_2_^•−^)	Tumor imaging	[196]
DMSN‐DP@CM nanosystem	miRNA‐21	Seesaw	Entropy‐driven process	PA780 (increased)/PA725 (decreased)	10 × 10^−12^ mol L^−1^–100 × 10^−9^ mol L^−1^	11.69 × 10^−12^ mol L^−1^	miRNA‐21 detection	[198]

#### 3.2.1 “Internal Reference Detection” PA Probes

PA‐responsive probes or polymers coloaded with inert PA contrast agents in the same nanocarrier enable “internal reference detection” while assuring spatial colocalization of both PA contrast agents.^[^ ^177^ ^]^ Typically, gold NPs, small molecule organic probes, and CuS nanomaterials serve as internal reference materials,^[^ ^178^ ^]^ whereas small responsive molecule organic probes and semiconductor polymers serve as responsive materials.

**Figure 10:**
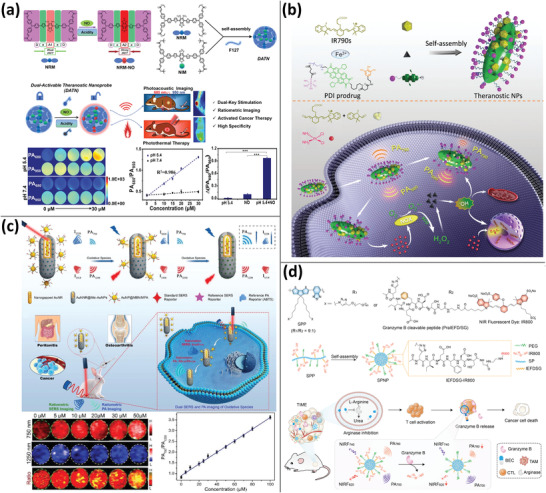
“Internal reference detection” for ratiometric PA imaging. a) By incorporating weak electron sensors into a D–A–D chromophore to create a NO/pH dual‐responsive ratiometric PA nanoprobe capable of discriminating tumors from normal tissues during PTT. Reproduced with permission.^[^ ^179^ ^]^ Copyright 2019, American Chemical Society. b) Simultaneous loading of ROS‐inert PDI and ROS‐responsive IR790 onto self‐assembled rod‐like NPs enabling PA imaging of ROS levels during tumor therapy. Reproduced with permission.^[^ ^181^ ^]^ Copyright 2018, John Wiley & Sons. c) Nanoprobes loading H_2_O_2_‐responsive HRP and its substrate ABTS, as well as H_2_O_2_‐inert AuNPs, enabling ratiometric PA imaging in tumor and inflammatory tissues. Reproduced with permission.^[^ ^184^ ^]^ Copyright 2020, John Wiley & Sons. d) Coupling of IR800 to the semiconductor polymer through a polypeptide cleavable by granzyme B. When cytotoxic T cells were stimulated followed by specific granzyme B cleavage, IR800 release resulted in a drop in PA signal while the PA signal of SPNs remained intact, allowing for the monitoring of granzyme B expression levels by PA imaging. Reproduced with permission.^[^ ^185^ ^]^ Copyright 2020, John Wiley & Sons.

Teng et al. developed NO/pH dual‐responsive ratiometric PA nanoprobes to enhance tumor tissue identification specificity. They initially incorporated a weak electron acceptor into a D–A–D type chromophore and then the weak electron receptor turned into a strong electron receptor when oxidized by NO in an acidic environment. This transition resulted in a considerable increase in the PA signal at 680 nm but did not affect the PA signal at 950 nm using an inert contrast agent of identical structure. The new probe generated a larger PA signal (9.8 times that of the NO‐responsive probe and 132 times that of the pH‐responsive probe), allowing for greater thermotherapeutic differentiation of tumor and normal tissue (**Figure** **10**a).^[^ ^179^ ^]^

Coloading catalytically active nanoenzymes with both responsive and inert probes amplified the signal ratio and increased sensitivity.^[^ ^180^ ^]^ Yang et al. coupled cisplatin‐activated nicotinamide adenine dinucleotide phosphate oxidase to PDI by PEG and polyphenols covalently bonded to Fe^3+^ at both ends of ROS‐inert PDI. The resulting compounds were then combined to form rod‐like NPs that loaded the ROS‐responsive dye IR790s. When the nanoprobes were exposed to ROS, their PA signal at 790 nm corresponding to IR790s reduced, their PA signal at 680 nm stayed unchanged, and the ratio of PA signals at 680/790 nm rose linearly with increasing H_2_O_2_ concentration. When PA was administered to tumor‐bearing animals, the ratio of PA signal might be used to determine the therapy effect (Figure 10b).^[^ ^181^ ^]^ Dhada et al. employed AuNRs as inert PA probes loaded with the ROS‐sensitive IR775c to monitor MSC activity. Degraded MSCs might be identified by comparing the ratio of PA signals at 790/900 nm (IR775c/AuNRs).^[^ ^182^ ^]^ Huang et al. created pH‐sensitive polyaniline‐coated gold nanotriangles to perform a broad range test for stomach acid, demonstrating a linear reduction in the ratio of PA signals at 790/1200 nm when the pH climbed from 1 to 8.^[^ ^183^ ^]^ Li et al. then created a core satellite nanoprobe for dual detection of H_2_O_2_ in vivo using SERS and PA. When in contact with H_2_O_2_, the HRP loaded in this system oxidizes ABTS to produce a strong absorbance at 750 nm, whereas the PA signal at 1250 nm of nanogapped AuNRs remained unchanged, and by comparing the PA signals at the two wavelengths, the H_2_O_2_ in rabbit osteoarthritis could be detected via PA imaging (Figure 10c).^[^ ^184^ ^]^

SPNs were also planned to be utilized as PA probes for “internal reference detection”. Granzyme B is required for the activation of cytotoxic T cells. Zhang et al. colinked a cleavable fragment of granzyme B to IR800 and subsequently to the surface of SPNs via a PA signal at 700 nm. When granzyme B was present, the polypeptide was cut off and IR800 was released, resulting in a reduction in the IR800‐corresponding PA signal at 760 nm, but no change in the PA signal at 700 nm by SPNs. The expression level of granzyme B might be determined in 4T1 tumor‐bearing mice by measuring the ratio of PA signals at 700/760 nm (Figure 10d).^[^ ^185^ ^]^ Yin et al. synthesized ClO^−^ degradable amphiphilic semiconductor polymers around which ClO^−^‐inert NIR775 was wrapped using the nanoprecipitation technique. When exposed to ClO^−^, the semiconductor polymers deteriorated, leading to a reduction in the PA signal at 680 nm, whilst the PA signal of NIR775 remained unaffected, allowing for quantitative detection of ClO^−^.^[^ ^186^ ^]^ Ma et al. employed an O_2_^•−^ inert semiconductor polymer as an internal reference, and nanoprecipitated the O_2_^•−^ responsive probe in DSPE‐PEG. When NPs were administered intravenously, they circulated to the location of atherosclerosis, resulting in an elevated PA signal at 690 nm, while the PA signal at 800 nm remained unaltered, allowing for higher ratio imaging of atherosclerosis.^[^ ^187^ ^]^ For the quantitative detection of ONOO^−^, Pu's group constructed organic semiconducting nanoprobes doped with boronate‐caged boron‐dipyrromethene dye. Due to the fact that the probe not only tended to produce conjugate acid at acidic pH, but also reacted to H_2_O_2_, the probe's specificity and sensitivity for the ONOO^−^ response were enhanced by adding bulky borane to the solution (buffering the low pH effect by Lewis acid–base reaction and acting as an inert shield against H_2_O_2_). When reacting with ONOO^−^, its maximum absorbance wavelength was redshifted from 645 to 745 nm, and the ratio of PA signals at 750/680 nm rose linearly as ONOO^−^ concentration increased.^[^ ^188^ ^]^

#### 3.2.2 “Seesaw Detection” PA Probes

By comparing PA signals excited at various wavelengths, ratiometric PA probes are utilized to identify chemicals. Due to the fact that PA detection is affected by certain factors such as contrast agent concentration, contrast agent distribution, probe loading, and artifacts, adding another wavelength PA signal to a single‐wavelength PA probe effectively eliminates environmental and instrument systematic errors and improves detection accuracy and precision, and thus these detective PA probes were defined as “seesaw detection” probes. There are four primary design options for “seesaw detecting” probes.

##### Active Probes Encapsulation

**Figure 11:**
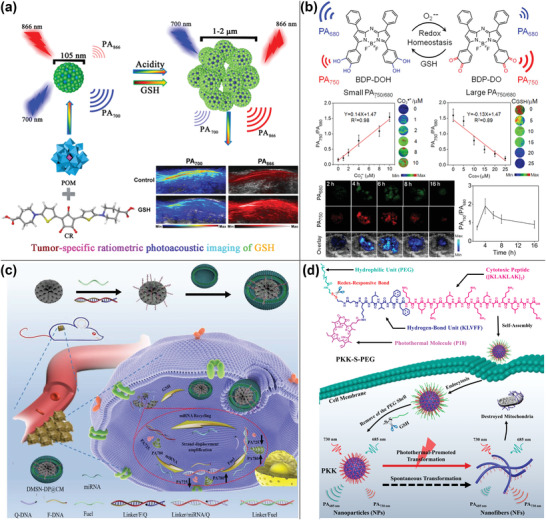
“Seesaw detection” for ratiometric PA imaging. a) GSH detection using croconaine dye and POM constructed nanomaterials. Reproduced with permission.^[^ ^189^ ^]^ Copyright 2019, American Chemical Society. b) PA imaging probes with reversible O_2_^·−^ and GSH reduction for monitoring tissue redox state through phenolic hydroxyl groups introduced into the BODIPY structure. Reproduced with permission.^[^ ^196^ ^]^ Copyright 2019, John Wiley & Sons. c) Dendritic mesoporous SiO_2_ NPs loading DNA‐PA probes and GSH‐responsive DNA fuel strands for the detection of miRNA‐21 in tumor tissues using PA imaging. Reproduced with permission.^[^ ^198^ ^]^ Copyright 2019, American Chemical Society. d) Redox‐responsive polymer–peptide conjugates were used to induce the development of nanofibers in the redox milieu of tumor tissues in order to provide PTT and ratiometric PA imaging of tumors. Reproduced with permission.^[^ ^200^ ^]^ Copyright 2020, American Chemical Society.

The ratiometric PA detection of pH in tumor tissues was accomplished by wrapping a commercial probe directly around it. Tang et al. assembled nanoplatforms using a GSH‐responsive croconaine dye and POM. GSH reduction dramatically decreased the absorbance of the croconaine dye at 700 nm, while GSH reduction progressively dissolved the POM cluster, resulting in a rise in absorbance at 866 nm. The ratio of PA signals at 866/700 nm rose linearly when GSH concentration was increased (**Figure** **11**a).^[^ ^189^ ^]^

##### Shift in Absorbance Wavelength

By altering the PA contrast agents, the maximum absorbance may be altered when they react with target molecules, and detection can be accomplished by comparing the PA signal at the maximum absorbance wavelength before and after the interaction. By altering PA contrast agents such as anthocyanines, BODIPY, and SPNs, the researchers developed ratiometric PA probes for the detection of a variety of compounds. The intermediate active site of cyanine dyes is often utilized to attach different responsive groups, resulting in a shift in their maximum absorbance wavelength due to structural changes after the reaction. For example, selenol‐responsive bis(2‐hydroxyethyl) disulfide was covalently linked to the intermediate active site of NIR cyanine dyes used to detect selenol in autoimmune hepatitis.^[^ ^190^ ^]^ Yin et al. substituted the active chlorine in IR806 with pyridine dithioethylamine, a GSH‐responsive molecule. In the presence of GSH, the disulfide bond was cleaved and reduced to sulfhydryl groups, which then replaced the secondary amine to form thio‐substituted IR806. After the reaction, the maximal absorbance wavelength of this probe was redshifted from 658 to 820 nm, and the ratio of PA signals at 820/658 nm rose linearly with the GSH concentration and peaked after 4 h of injection in tumor‐bearing animals in vivo.^[^ ^191^ ^]^ Liu et al. then enclosed the methylmercury (MeHg^+^)‐responsive hCy7 dye in the lipid bilayer of liposomes, leveraging MeHg^+^’s lipid solubility to react more readily with the hCy7 dye, resulting in a linear increase in the ratio of PA signals at 860/690 nm with increasing MeHg^+^ concentration, and successfully detected MeHg^+^ in zebrafish.^[^ ^192^ ^]^ Diabetes‐induced liver damage is intimately tied to the polarity of the endoplasmic reticulum in liver tissue. Xiao et al. developed a polarity detecting probe based on cyanine dyes, in which a tertiary amine served as an electron donor and a difluoroborate served as an electron acceptor, respectively, and the long‐conjugated system exhibited NIR absorbance. The ratio of PA signals at 700/800 nm reduced dramatically as ambient polarity increased, allowing for the detection of solution polarity using PA imaging.^[^ ^193^ ^]^

Additionally, BODIPY is often utilized as an “always‐on” PA contrast agent and as the foundation for the development of ratiometric PA probes.^[^ ^194^, ^195^ ^]^ Zheng et al. introduced phenolic hydroxyl groups into the structure of BODIPY, and its structure changed between phenolic hydroxyl groups and quinone‐like structures under the reversible action of O_2_^·^^−^ and GSH, resulting in a redshift of its maximum absorbance from 680 to 750 nm, with an increasing ratio of PA signals at 750/680 nm with increasing O_2_^·^^−^ concentration and decreasing with increasing GSH concentration, enabling reversible detections (Figure 11b).^[^ ^196^ ^]^

##### Method of Base Pairing

DNA and RNA double‐stranded structures with adjustable distance and complementary paired base sequence specificity are often employed to produce resonance energy transfer‐based fluorescence detecting systems, and researchers have used the technique for ratiometric PA imaging.^[^ ^197^ ^]^ Zhang et al. developed ratiometric PA probes loaded with DNA‐PA probes and GSH‐responsive DNA fuel strands to detect mouse tumor‐associated miRNA‐21. When the probe was introduced into tumor cells, it used hybridization to target miRNA‐21, preventing resonance energy transfer between IRDye 800CW and IRDye QC‐1, resulting in a decrease in PA signal at 725 nm and an increase in PA signal at 780 nm; additionally, when GSH in tumor cells triggered DNA fuel strand release, it replaced the already hybridized miRNA‐21 and amplified the signal, achieving PA detection of miRNA‐21 (Figure 11c).^[^ ^198^ ^]^

##### Responsive Chemical Bonds Cleavage to Form Aggregation

Through the use of a responsive linker, the “always‐on” PA contrast agents are connected to an amphiphilic polymer (e.g., GSH‐responsive disulfide bonds or enzyme‐cleavage polypeptide fragment). The disulfide bonds are cleaved by enzymes or GSH, causing the PA contrast agent to aggregate in tumor tissue, where the elevated PA signal may be observed for semiquantitative assembly detection. Wang's group constructed a PA probe from three components: purpurin 18, a cathepsin E‐responsive peptide, and a urokinase plasminogen activator receptor (uPAR)‐targeting peptide. Purpurin 18 could be formed in pancreatic cancer tumor cells after Cathepsin E cleavage of its response peptide, and the aggregation efficiency could be tracked via ratiometric PA imaging. Meanwhile, ratiometric PA imaging may be used to reduce the effect of concentration on the monitoring of aggregation efficiency measurement.^[^ ^199^ ^]^ The same group used a similar ratiometric PA probe to monitor the assemblies, which consisted of four components: a therapeutic peptide, a hydrogen‐bonding peptide, a hydrophilic PEG containing disulfide bonds, and purpurin‐18. In a physiological environment, the amphiphilic NPs self‐assembled into spherical NPs. When PEG was cleaved by GSH after entering tumor tissue, the hydrogen‐bonded peptide induced the remaining components to aggregate into nanofibers that could more easily interact with the mitochondrial membrane, and purpurin‐18 aggregation exhibited a stronger NIR photothermal effect, which further promoted nanofiber formation upon NIR laser irritation. This mechanism results in a reduction of PA signal at 685 nm and an increase of PA signal at 730 nm, allowing ratiometric PA imaging in vivo (Figure 11d).^[^ ^200^ ^]^ Miki et al. used a matrix metalloprotease‐2 (MMP‐2)‐responsive PLGLAG polypeptide to link PEG at an axial position in the plane of aluminum and silicon naphthalocyanines. When PEG chains were cut off specifically for contact with MMP‐2, aluminum naphthalocyanines formed H‐aggregates (face to face parallel aggregation) through a strong *π*‐interaction, but the interaction inside Si‐naphthalocyanines was weak owing to the larger ligand. The creation of H‐aggregates increased the PA signal at 680 nm and decreased at 760 nm, which might be utilized to monitor MMP‐2 in vivo.^[^ ^201^ ^]^

## 4 Conclusions and Perspective

Due to its excellent resolution, deep tissue penetration, and nonionizing radiation, PA imaging has garnered considerable interest and is employed in the diagnosis of illnesses, especially in the early stages. Unlike CT, MRI, and other imaging modalities, PA imaging is based on the laser absorbance of endogenous or exogenous contrast agents. The development of exogenous contrast agents not only compensates for the low absorbance intensity and low S/N ratio of endogenous contrast agents (e.g., oxyhemoglobin and deoxyhemoglobin, melanin, lipids), but also enables targeted imaging, multimodal imaging, and integration of diagnosis and treatment through the precise design of exogenous contrast agents. We outline the evolution of exogenous PA contrast agents during the last five years from a comprehensible standpoint and classify them into two categories: “always‐on” and “turn‐on”. “Always‐on” PA contrast agents rely on specific accumulation at the lesion site and have a limited S/N ratio; “turn‐on” PA contrast agents may respond to differences in the lesion microenvironment or to the specific expression of certain enzymes, and their intensity may be collected for detecting disease factors with a higher S/N ratio.

Despite the introduction of several PA contrast agents, issues are remained, and PA imaging is not flawless. In comparison to CT, MRI, positron emission tomography (PET), and other imaging modalities, PA imaging is unable to image the whole‐body or deeper tissue (the penetration depth of PA imaging is about 7 cm). To compensate for this disadvantage, a combination of whole‐body imaging contrast agent and PA contrast agent is required.^[^ ^110^ ^]^ Whether using endogenous or exogenous contrast agents, PA imaging must depend on the contrast agents’ photothermal conversion to create mechanical waves in order to detect the acoustic signal, which may not give sufficient information for weak light‐absorbing tissue. Additionally, although PA imaging can acquire vascular images, it can only produce fuzzy deep vascular images or vivid epidermal vascular images,^[^ ^202^, ^203^, ^204^, ^205^, ^206^ ^]^ which are not comparable to ultrasound, CT, MRI, or digital subtraction angiography.

Second, it is necessary to investigate the metabolism and long‐term safety of exogenous PA contrast agents in vivo. Exogenous NPs are recognized and captured by the immune system following intravenous injection, and a significant accumulation in the reticuloendothelial system, liver, and spleen is observed. Further investigation of the cumulative toxicity of some inorganic materials to major organs and their metabolism is required. Although advancements have been made by manufacturing NPs with smaller diameters (<5.5 nm)^[^ ^34^ ^]^ that can be flushed via the kidneys or by including biodegradable chemical bonds into SPNs, the particular metabolic routes in vivo and their ability to be totally eliminated remain unknown.

Once again, the contrast agent's qualities are improved. Currently, research on PA imaging contrast agents has been mostly focused on the NIR‐I region, but since its maximum permissible exposure intensity is lower than that of the NIR‐II region, it is critical to develop PA imaging contrast agents for use in the NIR‐II region.^[^ ^207^, ^208^ ^]^ According to the Jablonski energy level diagram,^[^ ^83^ ^]^ increasing nonradiative relaxation via aggregation‐induced fluorescence quenching is one of the most preferred strategies for enhancing PA signal, but this strategy results in the loss of original fluorescence signal, and fluorescence imaging, PDT, and PA signal are all in competition and can not be enhanced concurrently, and a situation is required immediate resolution. In comparison to “always‐on” contrast agents, “turn‐on” contrast agents may be initiated by the lesion site's unique physiological milieu or by highly expressed enzymes. The ideal “turn‐on” PA contrast agent will have a high sensitivity, a high specificity, and a high S/N ratio. The “single‐wavelength detection” PA probes responded to a single‐wavelength signal, which is easily affected by changes in the surrounding environment and the instrument itself; in particular, those PA probes whose PA signal intensity decreased as the detected substance increased demonstrated limited detective sensitivity.^[^ ^209^ ^]^ The “single‐wavelength detection” PA probes used a fixed wavelength as a reference, whereas the “seesaw detection” PA probes compensated for the error introduced by the surrounding environment and instrument by detecting the ratio of two wavelengths, resulting in increased sensitivity, specificity, and S/N ratio. Both “single‐wavelength detection” and “seesaw detection” PA probes are limited in their tissue penetration to the NIR‐I region. As a result, developing “turn‐on” NIR‐II PA probes may prove to be a useful strategy for increasing their detective sensitivity and accuracy. Additionally, several chemicals that are abundant at the lesion site (e.g., GSH) accumulate in normal tissues as well. Certain probes are unable to discriminate between lesioned and normal tissues, particularly those around the tumor. Although researchers have created “dual response” probes to compensate for these shortcomings, more efforts are necessary.^[^ ^163^ ^]^ The detection of pH at the lesion site currently relies on two approaches (protonation resulting in changes in absorbance or aggregation of AuNPs), both of which are sensitive to a narrow pH range (typically pH 5–7) and are therefore only applicable to tumor tissue or other mildly acidic physiological environments, whereas probes with a broader pH range are understudied.^[^ ^151^, ^210^ ^]^ Although “turn‐on” PA probes may compensate for the loss of fluorescence detection in terms of tissue penetration, their detection limits remain greater than those of fluorescence detection, implying that the development of more sensitive PA probes is also a critical future path. When bioactive NPs interact with proteins, cells, and tissues in vivo, they can generate biological responses, and PA imaging based on endogenous contrast agents can reflect physiological responses. The development of bioactive PA probes is capable of altering the physiological microenvironment (e.g., improving tissue hypoxia or lipid metabolism), and will allow not only dynamic monitoring of physiological changes, but also monitor the distribution of the exogenous contrast agent itself.^[^ ^139^ ^]^

Finally, while exogenous PA contrast agents have been created to provide excellent imaging, PA imaging based on physiological features should be emphasized as well. For example, PA imaging based on oxyhemoglobin/deoxyhemoglobin might be used in vessel and microvessel imaging as well as blood oxygen saturation monitoring, both of which have been used to diagnose illnesses such as breast cancer and arthritis.^[^ ^211^, ^212^ ^]^ Particularly, Lei et al. altered the hypoxic microenvironment of tumors using nanozymes with cyclic cascade catalysis. They conjugated glucose oxidase on the surface of high peroxidase‐like 2D PdMo bimetallic nanosheets, while the intrinsic LSPR of palladium‐based nanomaterials provided a photothermal effect to further facilitate this catalytic activity. 3D multispectral imaging could be used to evaluate oxyhemoglobin/deoxyhemoglobin levels in tumor tissue.^[^ ^213^ ^]^ Wu et al. utilized PA imaging to track the distribution of NIR‐797‐labeled thermosensitive dendrimers within tumors as well as to monitor blood oxygen saturation to characterize tumor hypoxia,^[^ ^214^ ^]^ and the same strategy was also used to monitor tumor hypoxia during treatment with anthraquinones.^[^ ^215^ ^]^ Furthermore, dual PA imaging of hemoglobin and melanin can be utilized to diagnose ocular diseases. The eyes are more susceptible to light irritation than other organs, and their maximum exposure dose is restricted.^[^ ^216^ ^]^ Thus, the use of exogenous contrast agents in ocular illnesses appears to be on the horizon. Furthermore, lipid metabolism is critical in disorders like obesity, and the development of exogenous PA probes for such conditions is still in its early stages.

In conclusion, PA imaging as a nonionizing and radiation‐free imaging technique has gained increasing interest from academics and clinicians. It has the ability to give not only qualitative information about illnesses, but also to aid in theranostic, and hence is projected to become a possible diagnostic imaging tool as well as aid in the treatment of diseases.

## Conflict of Interest

The authors declare no conflict of interest.
